# Towards industrial biological hydrogen production: a review

**DOI:** 10.1007/s11274-023-03845-4

**Published:** 2023-12-07

**Authors:** G. M. Teke, B Anye Cho, C. E. Bosman, Z. Mapholi, D. Zhang, R. W. M. Pott

**Affiliations:** 1 Department of Chemical Engineering, Stellenbosch University, Stellenbosch, South Africa; 2 Department of Chemical Engineering, University of Manchester, Manchester, UK

**Keywords:** Biohydrogen production, Biohydrogen modelling, Bioreactor configuration, Dark fermentation, Photofermentation, Techno-economic, Life cycle assessment

## Abstract

Increased production of renewable energy sources is becoming increasingly needed. Amidst other strategies, one promising technology that could help achieve this goal is biological hydrogen production. This technology uses micro-organisms to convert organic matter into hydrogen gas, a clean and versatile fuel that can be used in a wide range of applications. While biohydrogen production is in its early stages, several challenges must be addressed for biological hydrogen production to become a viable commercial solution. From an experimental perspective, the need to improve the efficiency of hydrogen production, the optimization strategy of the microbial consortia, and the reduction in costs associated with the process is still required. From a scale-up perspective, novel strategies (such as modelling and experimental validation) need to be discussed to facilitate this hydrogen production process. Hence, this review considers hydrogen production, not within the framework of a particular production method or technique, but rather outlines the work (bioreactor modes and configurations, modelling, and techno-economic and life cycle assessment) that has been done in the field as a whole. This type of analysis allows for the abstraction of the biohydrogen production technology industrially, giving insights into novel applications, cross-pollination of separate lines of inquiry, and giving a reference point for researchers and industrial developers in the field of biohydrogen production.

## Introduction

The world’s growing population, rapid urbanization, and industrialization have resulted in unprecedented energy demands. For instance, the global energy consumption was projected in the literature (IEA 2022; Sharma et al. 2022) to reach 20,000 million of tonnes oil equivalent (Mtoe) in 2050 from the 14,000 Mtoe in 2018 and 900 Mtoe in 1990. For just a 60-year period, the energy demands are extremely high to be solely met by non-renewable means such as coal, and natural gas. Furthermore, these non-renewable sources are classified as unsustainable options due to the dwindling fossil fuel supplies and anthropogenic activities (i.e., CO_2_ and other greenhouse gas emissions) triggering global warming, energy shortages and environmental degradation.

Conversely, renewable sources such as solar, wind, ocean, geothermal, and bioenergy are growing sectors, with bioenergy projected to account for more than half of the world’s energy needs by 2040 (Pandey et al. 2023). Among the bioenergy fuels like biodiesel, bioethanol, biomethane, and biohydrogen, biohydrogen is considered a viable alternative fuel due to four main reasons, namely (1) zero harmful emissions (i.e., it burns with O_2_ to release energy and H_2_O), (2) high calorific value of 122 MJ kg^−1^ (Yahaya et al. 2022) (i.e., higher than 29.2 MJ kg^−1^ for bioethanol (Sharma et al. 2023) and 50.0 MJ kg^−1^ for biomethane (Sharma et al. 2023), although a limitation is that densities of hydrogen are commonly low), (3) ambient synthesis (i.e., relatively low energy input requirements) (Anye Cho et al. 2021b), and (4) the potential for use in bioremediation (i.e., biosynthesized from waste materials facilitating waste recycling) (Anye Cho et al. 2021b). Although the global hydrogen demands, *circa* 90 Mt in 2020 (IEA 2022), have been met almost entirely by conventional methods utilizing the reformation of fossil fuels, production from renewable means and integration into bioremediation are attractive.

Even so, the commercialization of biologically produced hydrogen hinges upon innovation as the present production capacities are relatively low. Therefore, cumulative research over the decades has been tilted towards the innovations in (1) technical strategies, (2) production systems and configurations, (3) mathematical methods for modelling and optimizing the production systems, and (4) techno-economic and life cycles assessment, for informed investment decision making. The former [i.e., (1)] pertains to investigations into biophotolysis (direct and indirect), photo-fermentation, and dark fermentation. These strategies exist as a manifestation of the taxonomically diverse metabolic repertoire of isolated biohydrogen producing microorganisms. For example, whilst hydrogen is biosynthesized in the dark by some microbial species, others have additional physical/ biochemical requirements such as light, water, absence of oxygen, and catalyzing enzymes like hydrogenase and nitrogenase in order to produce hydrogen as a product. Mild changes in these biotic and abiotic parameters may trigger biohydrogen synthesis through yet to be identified metabolic pathways, justifying the significant research generated over the decades. To ensure this review is up-to-date, the over 2500 research articles published during the last decade (i.e., 2013 to 2023) on the various biohydrogen production strategies, totaling over 200 isolated biohydrogen producing microorganisms were summarized in the Biological Hydrogen Production Strategies section of this review. The state-of-the-art in investigating biohydrogen yields revealed a shift from solo to co-cultures attaining increases of up to 46%.

Regarding the second [i.e., (2)], production systems are typically bioreactors which are set up in varying configurations to cater for the biological demands of the microbes being utilized and process economies of scale. A thorough search for research articles spanning over two decades (i.e., 2003 to 2023) led to 6 main bioreactors configurations demonstrated in the literature, namely: continuous stirred tank reactor, reaching 3000 L scale (Lu et al. 2019), up-flow anaerobic sludge blanket bioreactor demonstrated at 22 L scale for hydrogen production (although commonly used at much larger scale for wastewater treatment) (Akhbari et al. 2023), column bioreactor at 5 L scale (Ross and Pott 2020), horizontal tubular bioreactor reaching 3000 m^2^ area scale (Legrand et al. 2021), and hybrid bioreactors (e.g., membrane bioreactors, multistage bioreactors) at laboratory scale (Buitrón et al. 2019). Irrespective of the bioreactor configuration and scale, different operational modes (i.e., batch, fed-batch, or continuous), and mixing strategies (i.e., agitation, aeration, peristaltic pump) were explored. Therefore, for each of the above mentioned biohydrogen production strategies, the advances towards industrialization, associated strengths and weaknesses of each operational mode, mixing strategy and bioreactor configuration are reviewed in detail in the Bioreactors modes and configurations section in this review. Among them, bioreactors operated with immobilized hydrogen-producing microbes reported enhanced gas–liquid mass transport with reduced mixing, induced shear stresses, and alleviating light attenuation for photobioreactors, thus proffering higher biohydrogen productivities, and presents potential production systems for industrial consideration.

As per the third [i.e., (3)]: mathematical methods for modelling and optimizing the production systems featured over 50 published research articles covering techniques such as kinetic modelling (excluding the well-reviewed unstructured approaches elsewhere Wang and Wan 2009; Nath and Das 2011; Chezeau and Vial 2019; Yahaya et al. 2022)) and constraint-based modelling, Computational Fluid Dynamics (CFD) and machine learning, for the past two decades (i.e., 2003 to 2023). The advent of high-performance computing resources enabling the faster computation of problems previously deemed intractable, for instance, CFD multi-scale simulations of industrial bioreactors, has spurred research endeavors within this theme. Therefore, the Modelling of biohydrogen production systems section of this review details the current state-of-the-art, and examines the limitations hindering industrial applications. Previous trends on kinetic modelling of biohydrogen production were updated with models predicting across different bioreactor scales and configurations, of great industrial interest, as well as approaches for accelerating faster computations of CFD-coupled biohydrogen kinetic models.

The final aspect for consideration, [i.e., (4)], techno-economic (i.e., TEA) and life cycles (i.e., LCA) assessments, features over 30 published research articles detailing the costs [i.e., capital costs (CAPEX), operational costs (OPEX)] and revenues associated with the entire biohydrogen production process. Although often omitted in previous literature reviews, techno-economic and life cycle assessments drive decision making for investments into the biohydrogen sections. Therefore, the Techno-economic and life cycle assessment strategies section of the review paper summarizes the cost evaluations and projections over the last decade. Noticeably, a significant number of articles carried out TEA without LCA, thus warranting integrated TEA and LCA to fully realize the commercialization potential of biohydrogen.

Lastly, the challenges and opportunities of the various innovation directions [i.e., (1) to (4)] are discussed in Future Perspectives section of this review paper as the future perspective toward commercializing biohydrogen production.

## Biological hydrogen production strategies

### Overvie*w*

**Fig. 1:**
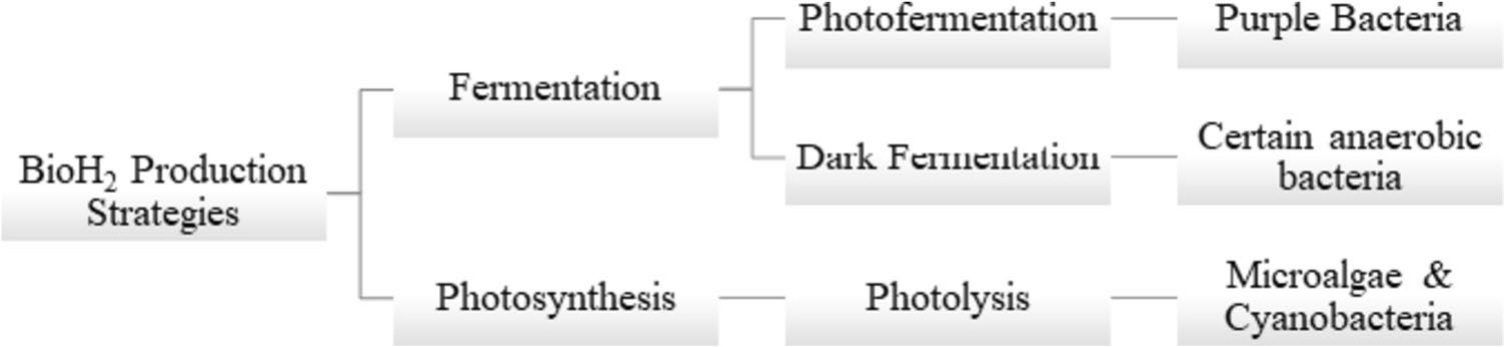
The biological hydrogen (bioH_2_) production strategies

The main biological hydrogen production strategies currently employed are (1) biophotolysis; (2) photo-fermentation; and (3) dark fermentation (Akhlaghi and Najafpour-Darzi 2020), as summarized in Fig. 1.

### Biophotolysis

Direct biophotolysis is the process whereby oxygenic photosynthetic microorganisms such as green algae and cyanobacteria (Ban et al. 2018; Akhlaghi and Najafpour-Darzi 2020) use light energy to convert water molecules into oxygen and molecular hydrogen, catalyzed by the hydrogenase enzyme: 2H_2_O → O_2_ + 2H_2_ (Hallenbeck and Ghosh 2009). The unicellular green alga, *Chlamydomonas reinhardtii*, is widely considered as the model organism for direct biophotolysis; however, several other algal species including *Chlorella vulgaris*, *Chlorella* sp*.* and *Scenedesmus obliquus* have also been employed for photolytic biohydrogen production (Winkler et al. 2002; Levin et al. 2004; Song et al. 2011; Ban et al. 2018; Javed et al. 2022). Cyanobacterial species most widely associated with photolytic biohydrogen production include *Anabaena variabilis*, *Nostoc punctiforme* and *Synechocystis* sp. (Javed et al. 2022).

Biohydrogen produced through biophotolysis is considered clean with low energy inputs; however, low energy conversion efficiencies are associated with direct biophotolysis partially due to oxygen-inhibition of the hydrogenase enzyme (Hallenbeck and Ghosh 2009). As per the stoichiometric splitting of a water molecule, a maximum hydrogen to oxygen ratio of 2:1 (mol/mol) can be obtained; however, for sustained hydrogen production in a biophotolysis system, oxygen should be maintained below a level of approximately 0.1%—severely limiting biohydrogen production (Melis et al. 2000; Bolatkhan et al. 2019).

Indirect biophotolysis is employed to enhance hydrogen production and circumvent the issue of oxygen-inhibition, thereby separating the evolved oxygen from the hydrogen gas (Eroǧlu and Melis 2011; Nanda et al. 2017). However, other literature-reported strategies to circumvent this issue include immobilizing microbes, nano-additives, oxygen removal through purging with inert gasses (Greenbaum 1982, 1988), the genetic modification of the algae to consume any produced oxygen (Ghirardi et al. 2000; Melis et al. 2000), sulfur removal from growth media to inhibit protein accumulation and cell growth, thus, lessening oxygenic activities (Melis et al. 2000), as well as photobioreactor configurations and designs (Javed et al. 2022; Suresh et al. 2023). Among these methods, the addition of nanoparticles and oxygen regulation through co-culturing with bacteria seem to be the most promising methods for enhanced biohydrogen productivity (Javed et al. 2022; Suresh et al. 2023). Through co-cultivation, hydrogen production by *Chlamydomonas reinhardtii* has been reported to increase by 5.3-fold and up to 14-fold using *Azotobacter chroococcum* (Xu et al. 2017) and *Bradyrhizobium japonicum* (Xu et al. 2016), respectively, notably reducing oxygen levels. Increases in biohydrogen production of up to 24%, 46% and 32% have been reported through co-cultivation of *Chlamydomonas reinhardtii* with *Escherichia coli*, *Pseudomonas stutzeri*, and *Pseudomonas putida*, respectively (Fakhimi and Tavakoli 2019). In addition to *Chlamydomonas*, photolytic hydrogen production has also been shown to be promoted in other algal–bacterial co-cultures—*Chlorella protothecoides*, *Chlorella pyrenoidosa* and *Scenedesmus obliquus* all individually partnered with the bacterial species *Pseudomonas* sp. (Ban et al. 2018).

As mentioned, the addition of nanoparticles also seems to notably affect photolytic hydrogen production. For example, silica nanoparticles have been utilized to enhance light uniformity, thereby increasing the biohydrogen productivity of *Chlamydomonas reinhardtii* by up to 23% during production (Giannelli and Torzillo 2012). Furthermore, *Chlamydomonas reinhardtii* cells encapsulated in sulfur-deprived TiO_2_ have similarly been demonstrated to double the efficiency of a photolytic hydrogen production system (Stojkovic et al. 2015). Furthermore, it is worth noting that the silica nanoparticle photobioreactor system had a working volume of 110 L, leaning towards pilot-scale operation and indicating the viability of scale-up of biophotolysis hydrogen production systems (Giannelli and Torzillo 2012). Nonetheless, the insurmountable oxygen-sensitivity challenges of biophotolysis hydrogen production systems have drifted the literature focus towards alternative biohydrogen production methods.

### Photofermentation

Photofermentative hydrogen production by anoxygenic bacteria is conducted primarily via the nitrogenase enzyme, which facilitates the conversion of organic carbon to biohydrogen (approx. 96% Pott et al. 2013; Bosman et al. 2022) and carbon dioxide, while fixing molecular nitrogen (McCully and McKinlay 2016; Xiao 2017). Photofermentative hydrogen is mostly produced through the Gram-negative prokaryotes, purple bacteria—the most commonly employed organisms being purple non-sulfur bacteria belonging to the class of *Alpha-proteobacteria* (Koku et al. 2003; Larimer et al. 2004). Biohydrogen producing species in the group of purple non-sulfur bacteria include *Rhodobacter spaeroides*, *Rhodobacter capsulatus, Rhodospeudomonas palustris*, and *Rhodospirillum rubrum*.

Laboratory-scale studies generally utilize a vast number of organic carbon substrates for biohydrogen production by purple non-sulfur bacteria, with organic acids and certain sugars typically being the preferred conventional substrates as seen in Table 1. Important to note, however, is that some purple non-sulfur bacteria species lack certain transport proteins, specifically glucose and fructose transporters necessary for sugar metabolism (Larimer et al. 2004). Furthermore, from a sequential dark and photofermentation perspective, acetic and butyric acid are typically most abundant in the effluent resulting from dark fermentation (which will be discussed in the Dark Fermentation section)—organic acids, which can be further utilized in photofermentation to maximize biohydrogen yield (Eroǧlu and Melis 2011; Rai and Singh 2016). Nonetheless, considering the environmental impact and high costs of pure substrates, many studies have shifted their focus towards the use of waste streams as the substrate (e.g., food/crop waste, and/or wastewater streams), as shown in Table 1 (Melitos et al. 2021)—aiming towards a circular bio-economy (Zhang et al. 2017; Pott et al. 2018; Uys 2019; Melitos et al. 2021). Photofermentative microbes have been demonstrated to produce biohydrogen from suitable waste streams at production rates comparable to conventional substrates—a key factor in measuring the viability of potentially using commercial waste streams for biohydrogen production.

**Table 1:** Examples of substrates used by purple non-sulfur bacteria for biohydrogen production via photofermentation

Microorganism	Substrate	Working volume (L)	H_2_ rate	References
Conventional substrates				
* Rhodopseudomonas palustris* NCIMB 11774	Acetic acid	0.5	~ 23 mL/g/h	Pott et al. (2013)
* Rhodopseudomonas palustris* sp.	Acetic acid	0.22	15.21 mL/L/h	Padovani et al. (2015)
* Rhodopseudomonas sphaeroides* strain A7	Acetic acid	0.025	31.54 mL/L/h	Wen et al. (2017)
* Rhodopseudomonas palustris* DSM 127	Acetic acid	0.6	3.9 mL/L/h	Hu et al. (2018)
* Rhodopseudomonas palustris* NCIMB 11774	Butyric acid	0.5	~ 20 mL/g/h	Pott et al. (2013)
* Rhodopseudomonas palustris* DSM 127	Butyric acid	0.6	19.9 mL/L/h	Hu et al. (2018)
* Rhodopseudomonas palustris* GCA009	Glucose	0.9	79.7 mL/m2/h	Wang et al. (2019)
* Rhodopseudomonas palustris* NCIMB 11774	Glucose	0.5	~ 28 mL/g/h	Pott et al. (2013)
* Rhodopseudomonas palustris* NCIMB 11774	Glycerol	1	3.8 mL/L/h	Bosman et al. (2022)
* Rhodopseudomonas palustris* NCIMB 11774	Glycerol	0.5	~ 31 mL/g/h	Pott et al. (2013)
* Rhodopseudomonas palustris* CGA009	Glycerol	0.5	16.2 mL/L/h	du Toit and Pott (2021)
* Rhodopseudomonas palustris* ATH 2.1.37	Glycerol	0.5	22 mL/L/h	du Toit and Pott (2021)
* Rhodopseudomonas palustris* NCIMB 11774	Lactic acid	0.5	~ 24 mL/g/h	Pott et al. (2013)
* Rhodopseudomonas palustris* DSM 127	Lactic acid	0.6	8.4 mL/L/h	Hu et al. (2018)
* Rhodopseudomonas palustris* NCIMB 11774	Malic acid	0.5	~ 15 mL/g/h	Pott et al. (2013)
* Rhodopseudomonas palustris* 420L	Malic acid	0.25	21.8 mL/L/h	Muzziotti et al. (2016)
* Rhodopseudomonas palustris* DSM 127	Malic acid	0.6	29.6 mL/L/h	Hu et al. (2018)
* Rhodopseudomonas palustris* DSM 123	Malic acid	1	7 mL/L/h	Basak et al. (2016)
Low-cost ‘waste’ substrates				
HAU-M1 consortium (*Rhodospirillum rubrum*, *Rhodobacter capsulatus* and *Rhodopseudomonas palustris*)	Apple waste	0.25	–	Lu et al. (2016)
*Rhodopseudomonas palustris* NCIMB 11774	Crude glycerol	0.5	34 mL/g/h	Pott et al. (2013)
*Rhodopseudomonas palustris* CGA009	Palm oil & soybean oil	–	–	Phongjarus et al. (2018)
*Rhodobacter sphaeroides* Consortium^a^	Wheat powder	0.25	–	Argun et al. (2008)
Consortium 1^b^	Corn stalk pith	0.22–0.32	96 mL/L/h	Jiang et al. (2016)
Consortium 2^c^	Corncob	0.1	133.7 mL/L/h	Zhang et al. (2014b)
Consortium 2	Sorghum stover	0.1	91.2 mL/L/h	Zhang et al. (2014b)
Consortium 2	Corn stover	0.1	88.5 mL/L/h	Zhang et al. (2014b)
Consortium 2	Rice straw	0.1	84.2 mL/L/h	Zhang et al. (2014b)
Consortium 2	Soybean stalk	0.1	74.1 mL/L/h	Zhang et al. (2014b)
Consortium 2	Cotton stalk	0.1	61.4 mL/L/h	Zhang et al. (2014b)
*Rhodopseudomonas palustris* 420L	Bread waste	0.1	1.96 mL/L/h	Zhang et al. (2014b)
*Rhodobacter sphaeroides* S10	Empty fruit bunch	1	51.63 mL/L/h	Palamae et al. (2018)

^a^Rhodobacter sphaeroides Consortium: *Rhodobacter sphaeroides* NRRL B-1727, *Rhodobacter sphaeroides* DSMZ & *Rhodobacter sphaeroides*-RV)

^b^Consortium 1: *Rhodospirillum rubrum*, *Rhodopseudomonas capsulata*, *Rhodopseudomonas palustris*, *Rhodobacter sphaeroides*, and *Rhodobacter capsulatus*

^c^Consortium 2: *Rhodospirillum rubrum*, *Rhodobacter capsulatus*, and *Rhodopseudomonas palustris*

### Dark fermentation

Dark fermentation is a light independent process during which microorganisms break down certain carbohydrate-rich substrates to generate molecular hydrogen and other by-products (Hallenbeck 2009; Hallenbeck and Ghosh 2009). Facultative or obligate anaerobic bacteria are most widely associated with dark fermentation – this includes species such as *Clostridium* spp., *Enterobacter* spp., *Enterobacter* spp. and *Bacillus* spp. (Levin et al. 2004; Wang et al. 2020).

Regarding the utilized substrates for dark fermentation, various sources summarized in column 2 of Table 2 are often implemented. However, with the view on industrialization and achieving a circular bio-economy, many studies have investigated dark fermentative biohydrogen production from ‘waste’ streams, similar to studies following the photofermentation route—such waste streams include complex organic compounds such as food waste, municipal waste, starch and lignocellulosic biomass (see Table 2). Generally, the most preferred substrates for biohydrogen through dark fermentation are monosaccharide (i.e., glucose) and disaccharide (i.e., sucrose) sugars, typically converted from cellulose and hemicellulose, amongst others (Łukajtis et al. 2018). However, the bottleneck of using naturally occurring cellulose and hemicellulose is the significant costs associated with pre-treatment of such substrates as they are not readily fermentable by most organisms (Nissilä et al. 2014; Łukajtis et al. 2018; Basak et al. 2020).

**Table 2:** Literature examples of substrates used for biohydrogen production via dark fermentation

Microorganism	Substrate	Working volume (L)	H_2_ rate	References
Conventional sugar substrates				
*Clostridium butyricum* INET1	Hexoses	0.1	–	Yin and Wang (2017)
*Clostridiaceae* & *Flexibacteraceae*	Glucose	2	640 mL/L/h	Oh et al. (2004)
*Clostridium* sp. 6A-5	Glucose	0.1	269.3 mL/L/h	Cai et al. (2013)
*Clostridia* sp.	Glucose	–	7.42 mmol/g_VSS_.h^a^	Lin and Chang (2004)
Mixed culture	Glucose	1.7	–	Fang and Liu (2002)
Mixed culture	Sucrose	4	0.105 mol/h	Chen et al. (2001)
*C. termolacticum*	Lactose	2	2.58 mmol/L/h	Collet et al. (2004)
Mixed culture	Xylose	0.9	7.3 mmol/L.h	Mäkinen et al. (2012)
*E. cloacae* IIT-BT 08	D-Xylose	2	3.48 mL/L/h	Kumar and Das (2000)
Low-cost 'waste' substrates				
Mixed culture	Cow waste	1	–	Yokoyama et al. (2007)
Mixed culture (incl. *Selenomonas* sp & *Clostridium* species)	Beverage wastewater	–	2292 mL/L/h	Sivagurunathan et al. (2015)
Mixed culture from WWTP	Rice winery wastewater	3	387.5 mL/VSS/h	Yu et al. (2002)
*T. thermosaccharolyticum*	Cellulose	–	12.08 mmol/h	Show et al. (2012)
Anaerobic sludge from baker’s yeast company	Waste-paper hydrolysate	0.15	–	Eker and Sarp (2017)
Anaerobic mixed microflora from sewage sludge digester	Untreated cellulose	6	–	Gadow et al. (2012)
Mixed cultures from cow manure	Grass silage	0.06	–	Pakarinen et al. (2008)

^a^*VSS* Volatile suspended solids

Mixed culture: Mostly *Citrobacter freundii*, *Clostridium acetobutylicum*, *C. freundii*, *Clostridium butyricum*, *Clostridium tyrobutyricum*

*WWTP* Wastewater treatment plant

While significant progress has been made with regards to identifying alternative, more cost-effective substrates for both photofermentative and dark fermentative biohydrogen production, generally, most waste streams still require pre-treatment. Pre-treatments include but are not limited to acid pre-treatment and/or enzymatic hydrolysis of crop wastes, for instance, to convert the lignocellulosic biomass into sugars readily consumable by the microorganisms, *i.e.* a hydrolysate (Nissilä et al. 2014; Basak et al. 2020). Furthermore, the use of waste substrates in practice also raises some important questions. With the view on industrialization, such waste streams will potentially have to undergo upstream sterilization on large scale for example, or the bacterial cells will have to be immobilized. However, these questions pertaining to industrialization significantly impact economic feasibility and practicality but are still primarily unanswered. Other important aspects of biohydrogen production from wastewater (Venkata Mohan 2009; Arimi et al. 2015; Gunasekaran et al. 2019; Preethi et al. 2019; Rajesh Banu et al. 2020; Dange et al. 2021; Feng et al. 2022; Qyyum et al. 2022)and solid waste (Kapdan and Kargi 2006; Bharathiraja et al. 2016; Yun et al. 2018; Keskin et al. 2019; Tian et al. 2019; Kamaraj et al. 2020; Srivastava et al. 2021; Ananthi et al. 2022) have been comprehensively reviewed elsewhere.

Generally speaking, biohydrogen production via dark fermentation is considered the best biological hydrogen production route due to high production rates, as seen in Table 2. However, despite the advantage of being a light-independent process, contrary to the photofermentative route, dark fermentative biohydrogen production is hampered by lower yields and the formation of large volumes of organic by-products—typically acetic and butyric acid (Hallenbeck and Ghosh 2009). In addition, dark fermentation also generates a large percentage of CO_2_, along with traces of CH_4_, CO and H_2_S, which further require downstream separation and purification (Nanda et al. 2017). Even so, several studies have been conducted on dark fermentative biohydrogen production within continuous bioreactors, enabling the potential for scale-up; however, low biohydrogen yields are associated with such processes (Sivagurunathan et al. 2016b)—below approximately 4 mol H_2_/mol substrate (Oh et al. 2004; Chen et al. 2012; Gadow et al. 2012; Mäkinen et al. 2012; Carrillo-Reyes et al. 2014; Sivagurunathan et al. 2014).

Other factors hindering commercialization of dark fermentative biohydrogen production include *inter alia*, inorganic inhibitors [e.g., heavy and light metal ions, ammonia, and gas typically present in wastewater, waste activated sludge and food waste (Li and Fang 2007)], organic inhibitors [e.g., volatile fatty acids Wang et al. 2008; Ciranna et al. 2014; Yang and Wang 2018), furan derivatives (Lin et al. 2015; Eker and Sarp 2017; Basak et al. 2020), and phenolic compounds (Lin et al. 2015)], bio-inhibitors [e.g., bacteriocins typically used as preservatives in food products (Gomes et al. 2016; Ohnishi et al. 2022) and thiosulfinates (Tao et al. 2020)] (Chen et al. 2021). Of these inhibiting factors, the Achilles’ heel of dark fermentation upscaling seems to be lactic acid bacterial contamination and, thus, inhibition by bacteriocins (Ohnishi et al. 2022). Although mitigating steps have been considered to circumvent the inhibition of biohydrogen production, the inhibition thresholds are system specific. Therefore, as we are moving towards industrialization and hence the use of waste substrates, more research is needed into the effects of inhibiting compounds in waste streams and their large-scale impact. That is, the economic feasibility of using waste streams containing such inhibitors for biohydrogen production, as the use of waste streams are currently still economically prohibitive (Basak et al. 2020).

## Bioreactors modes and configurations

**Fig. 2:**
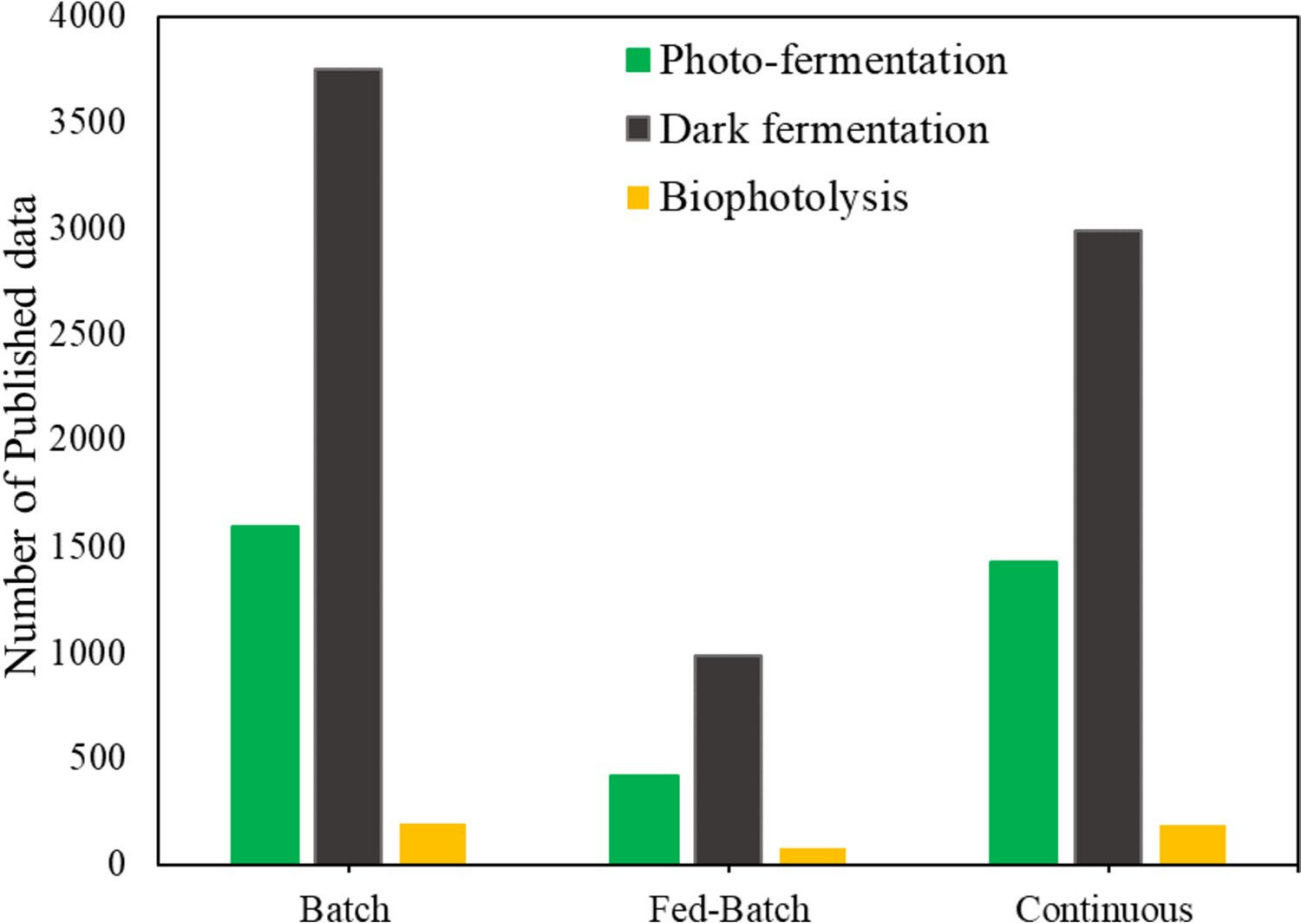
A summary of published data (from 2008–2023) based on the 3 modes of operation and the production routes of biohydrogen which include biophotolysis, photo-fermentation and dark fermentation

For an industrial process to be viable, the process should strive to achieve the maximum possible productivity and yield at the lowest possible cost, which can be driven by the physical configuration. Hence, bioprocess engineers need to examine and understand current production configurations in parallel with their different modes of operations to facilitate the implementation of systems which are robust and cost effective. Thus, this section presents the different modes of operation, in conjunction with their bioreactor configurations, used for biohydrogen production. Based on published literature, Fig. 2 presents the number of articles published in each of the three traditional routes (biophotolysis, photo- and dark fermentation) used to produce biohydrogen, based on the different modes of operation. From Fig. 2, it is evident that the majority of research focusses on photo- and dark fermentation and hence, this section will focus specifically on these two production strategies.

### Modes of operation used for biohydrogen production

#### Dark fermentation modes of operation

**Fig. 3:**
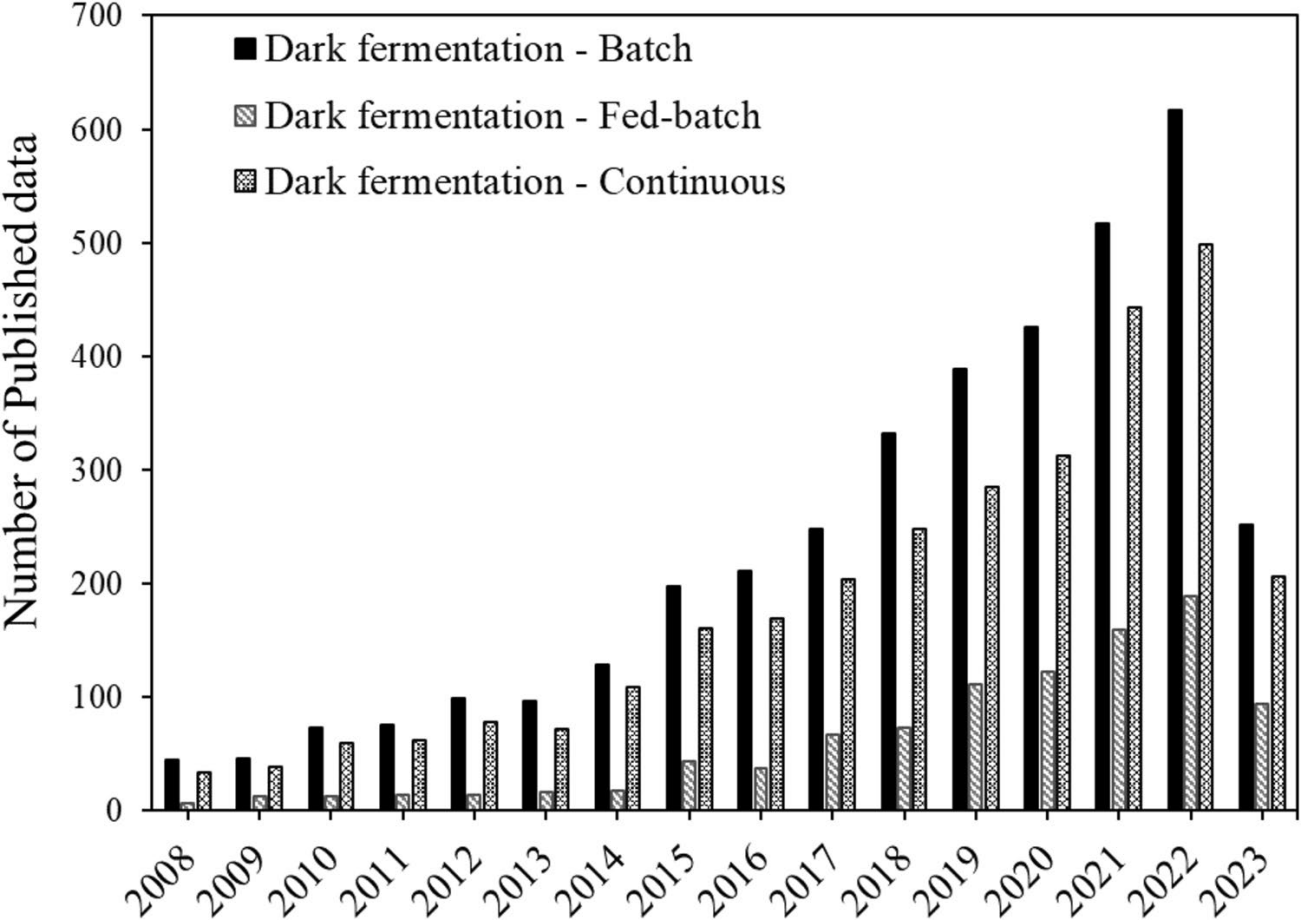
A summary of published data (from the year 2008 to mid-2023) of biohydrogen production based on dark fermentation modes of operation

Dark fermentation as route of biohydrogen production has seen the implementation of the batch, fed-batch, as well as continuous modes of operation, with a total of about 7750 articles published between the years of 2008 to 2023. From these three modes, dark fermentative biohydrogen has predominantly been produced in the batch mode of operation (Fig. 3). Studies on this have primarily been based on understanding the numerous species of microorganisms that can facilitate the process of producing biohydrogen or have been focused on investigating the effect of different substrates or operating parameters to enhance yield and productivity, as shown in several reviews (Argun and Kargi 2011; Azwar et al. 2014; Banu et al. 2021; Zhang et al. 2023a).

Therefore, batch mode, dominated by laboratory demonstration, has extensively been used to understand how to enhance biohydrogen generation. In terms of scalability, most of these studies have focused on laboratory-scale, ranging from 200 mL to 10 L.

Even though most of the dark fermentation biohydrogen research was conducted using batch mode, continuous mode follows suit in the number of research articles published, as shown in Fig. 3. Continuous mode should thus be regarded as a potential route towards increasing economies of scale industrially. However, similar to batch mode studies, most investigations have relied on investigating potential substrates, and microbial species, to better describe, understand and investigate yield and productivity (Patel et al. 2021; Lee et al. 2022). Nevertheless, continuous mode, in comparison to batch, depicts higher biohydrogen productivities throughout steady state operation (Buitrón et al. 2014; Kumar et al. 2016; Mikheeva et al. 2021). Additionally, in order to enhance production, these two modes of operation have both occasionally utilized immobilization techniques, however, productivity consistently remained higher during continuous mode as compared to batch mode (Keskin et al. 2018; Sekoai et al. 2018; Banu et al. 2021; Patel et al. 2021; Mikheeva et al. 2022).

When the fermentative organisms encounter high biomass and/or substrate concentrations, fed-batch (i.e., semi-continuous) mode may be advantageous in circumventing substrate/product or toxic compound inhibition, in comparison to batch and continuous modes (Escamilla-Alvarado et al. 2013; Ghimire et al. 2017; Chen et al. 2022). Also, in scenarios where the microbial metabolic rate requires alteration during fermentation, fed batch systems are ideal, since the metabolism can be indirectly controlled through substrate concentration by changing flowrates and compositions. This suggests that it could be the way forward for the scale-up of dark fermentation, as up to 50 L scale up for 30 days of continuous biohydrogen production has been reported (Kovalev et al. 2023).

In terms of scalability potential, dark fermentation has demonstrated the possibility of upscaling to producing units from 0.4–3000 L as pilot-scale setups (Krupp and Widmann 2009; Lu et al. 2019). However, this technique can be beneficial towards scaling laboratory experiments through an exploration of mathematical models as a proposed potential approach (Khanna and Das 2013; Albanez et al. 2016; Volpini et al. 2018), discussed in the Modelling of biohydrogen production systems section of the review.

#### Photofermentation modes of operation

**Fig. 4:**
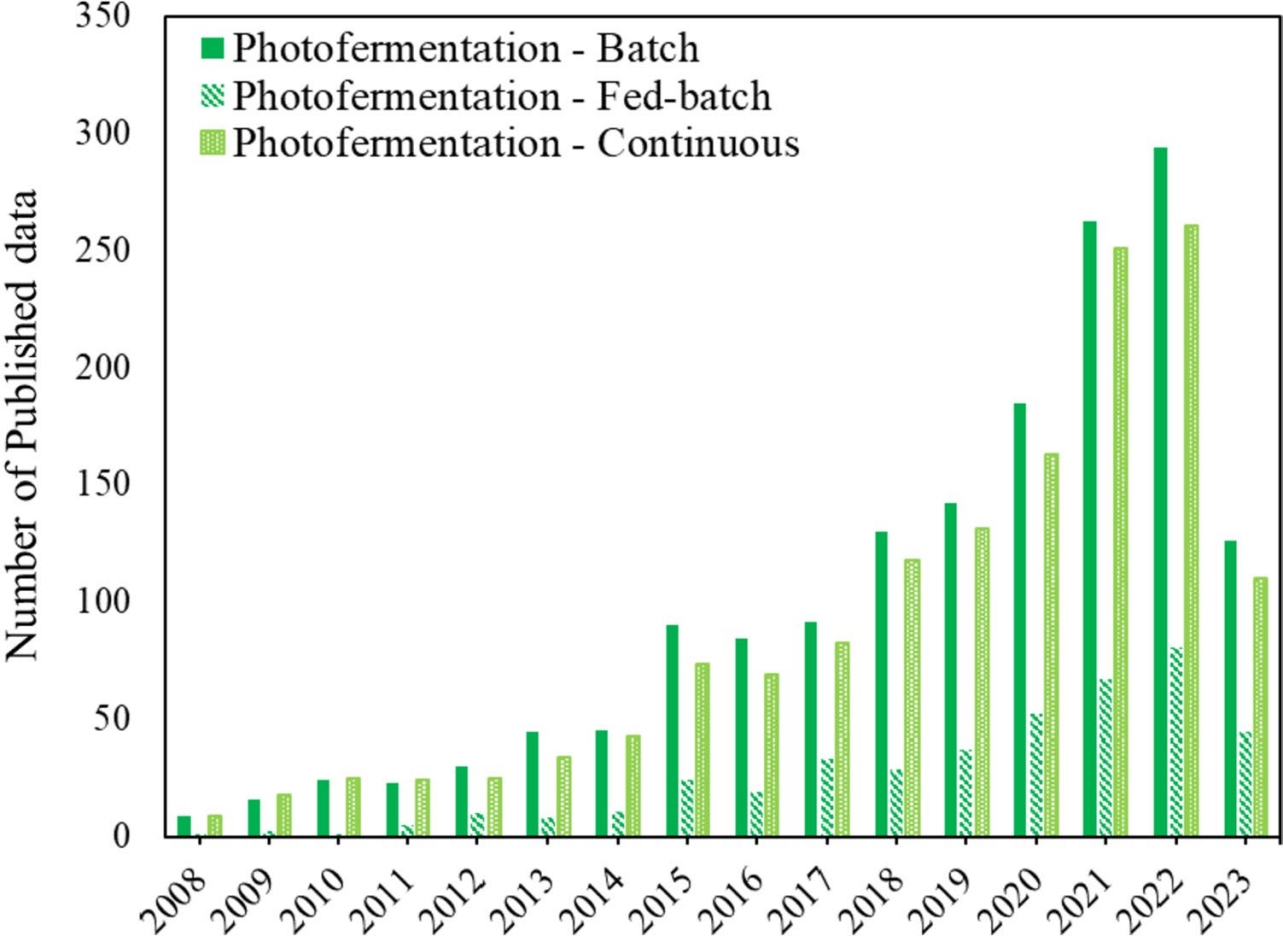
A summary of published data (from years 2008 to 2023) of biohydrogen production based on photo fermentation modes of operation

Similar to dark fermentation, a significant amount of photofermentation research has focused on the batch mode of operation (as shown in Fig. 4) with the studies in general varying micro-organism, operational parameters and substrates (Argun and Kargi 2011; Cheng et al. 2022). In terms of carbon sources used, the focus has primarily been on pure carbon sources kept in sterile fermentation media to investigate optimum conditions based on operating choices selected (Hitam and Jalil 2020; Cheng et al. 2022; Policastro et al. 2022). This is clearly a simplification of what would happen industrially, and studies on mixed, non-sterile, or non-pure substrates are less frequent.

Just like dark fermentation, photofermentative biohydrogen production has seen fewer studies using the fed-batch and the continuous modes of operation (as shown in Fig. 4), highlighting an area which requires further research if industrial scalability of this production strategy is to be evaluated. These studies investigated the effects of substrate, micro-organism used, and the variability of experimental conditions. These parameters were shown to significantly affect the biohydrogen yield and productivity (Oncel and Vardar-Sukan 2009; Basak et al. 2014; Hwang and Lee 2021; Policastro et al. 2022).

In terms of scalability, the majority of photo-fermentation projects are still conducted on laboratory-scale with volumes ranging between 100 mL and 10 L (see Table 1). Pilot-scale has been demonstrated with the combination of both dark- and photo-fermentation of a total capacity of 3000 L (Lu et al. 2020). Also, sequential dark- and photo-fermentation of a capacity of 30 L has been demonstrated (Nasr et al. 2015) making this combination an interesting option for system scalability, as well as substrate conversion efficiency.

Furthermore, most photo-fermentation biohydrogen production studies have been conducted under controlled indoor conditions (Pott et al. 2013; Du Toit and Pott 2020; Ross and Pott 2020; Mabutyana and Pott 2021; Bosman et al. 2022), with limited photo-fermentation studies under natural outdoor conditions (Boran et al. 2010; Gebicki et al. 2010; Adessi et al. 2012; Özkan et al. 2012). Therefore, investigations mimicking or under natural sunlight are necessary for scaling up and industrialization. However, outdoor biohydrogen production studies tend to be complex, due to the fluctuations in uncontrolled conditions, therefore, indoor studies could also implement artificial illumination resembling that of typical sunlight required by photosynthetic microbes (Uyar et al. 2007; Eroglu et al. 2010; Bosman et al. 2023b).

### Bioreactor configurations used for biohydrogen production

The chosen type and configuration of bioreactor used during biohydrogen production have been shown to affect substrate conversion efficiencies, and operability. Several bioreactor types have been demonstrated in the literature, namely: CSTR, upflow anaerobic sludge blanket, vertical tubular, horizontal tubular, flat panels, and hybrid. However, if industrial scalability is a focus, then the bioreactor configurations are still to be further investigated and optimized. This section discusses the widely used bioreactor configurations during dark- and photo-fermentation, based on their operations and scalability potential, coupled with some detail of their general design structure.

#### Continuous stirred tank reactor (CSTR)

**Fig. 5:**
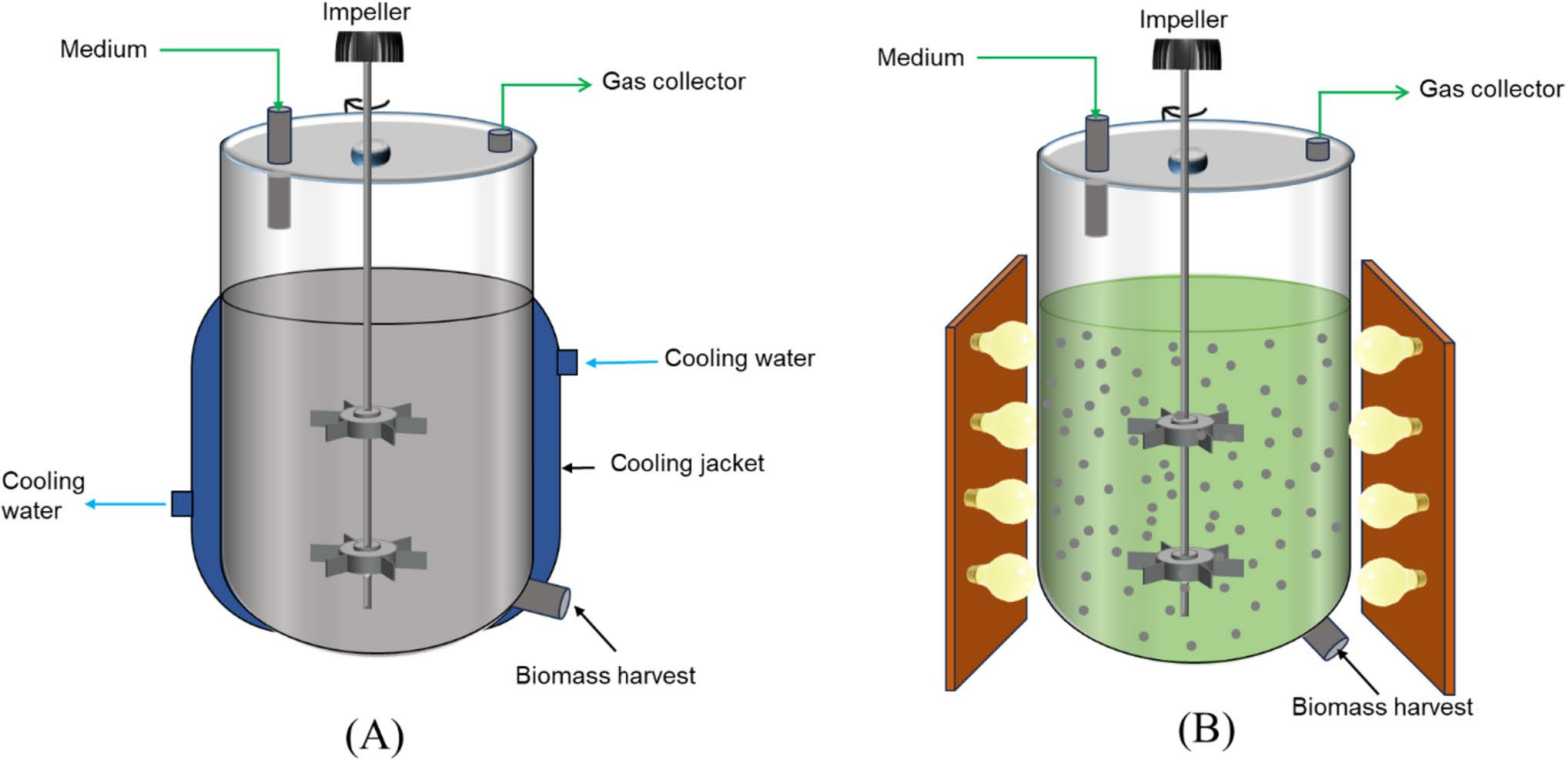
Continuous stirred tank bioreactor/Fermenter, **A** dark fermentation and **B** photo fermentation (lots of research done on magnetic stirred systems) modified from (Younesi et al. 2008; Castillo-Hernández et al. 2015)

CSTR configurations have commonly been utilized for dark- and photo-fermentation (and are well studied from the standard bioprocessing literature), as shown in Fig. 5, with some modifications for photofermentation in Fig. 5B. This configuration is notable for its excellent mixing, thereby permitting the microbes to generate biohydrogen from thoroughly mixed substrate. These hydrodynamic circumstances permit good substrate-to-microbial cells contact, thereby allowing for efficient microbial mass transfer and biohydrogen production (Brindhadevi et al. 2021), although the good mixing comes at the cost of high shear stresses, and the requirement for significant electricity input. In scenarios of limited mass transfer, controlled conditions could be employed in a sequencing batch reactor which are designed to provide adequate stirring with reduction in the shear strain formed on the biomass (Castillo-Hernández et al. 2015).

Several species, substrates, and operating conditions have been demonstrated to produce biohydrogen in a CSTR configuration. Additionally, microbial immobilization has also been employed in this configuration, as a strategy to improve the volumetric biohydrogen productivity (Canbay et al. 2018) and to separate hydraulic and solids retention times. However, again, the major challenge with the use of immobilization in CSTRs is the deformation or damage of the bead caused by the mixing impellers—this may be resolved by reducing the impeller speed or decreasing the bead size (Canbay et al. 2018; Keskin et al. 2018). As shown in other microbial production routes (Teke et al. 2021, 2022a), this configuration is promising should proper design optimization towards biohydrogen production be implemented. In terms of design optimization, two aspects in a CSTR configuration are of importance, that is, hydrodynamics and component mass transfer. The former is driven by aspects such as geometric configurations, flow pattern, mixing, turbulence, shear stress while the latter would involve the multi-phase component interaction. These two aspects have been carefully studied for a traditional or a modified CSTR configuration (Gakingo et al. 2020; Teke and Pott 2021; Teke et al. 2022b, c) and an adaptation into CSTR bioreactors for biohydrogen production could be of benefit.

Nevertheless, biohydrogen production in CSTR configurations has seen an improvement from laboratory-scale [e.g., 250–750 mL Erlenmeyer shake flask—Stavropoulos et al. 2016; Du Toit and Pott 2020, 2021; Mabutyana and Pott 2021)] to benchtop bioreactors [1 to 3 L—Nualsri et al. 2016; Hassan et al. 2019)] and semi-pilot plant bioreactors [10 L—(Sekoai and Gueguim Kana 2014)]. However, larger scalable production systems are still required when compared with the several conventional literature-reported bioprocessing production systems exceeding 2000 L working volumes.

#### Upflow anaerobic sludge blanket (UASB) bioreactor

**Fig. 6:**
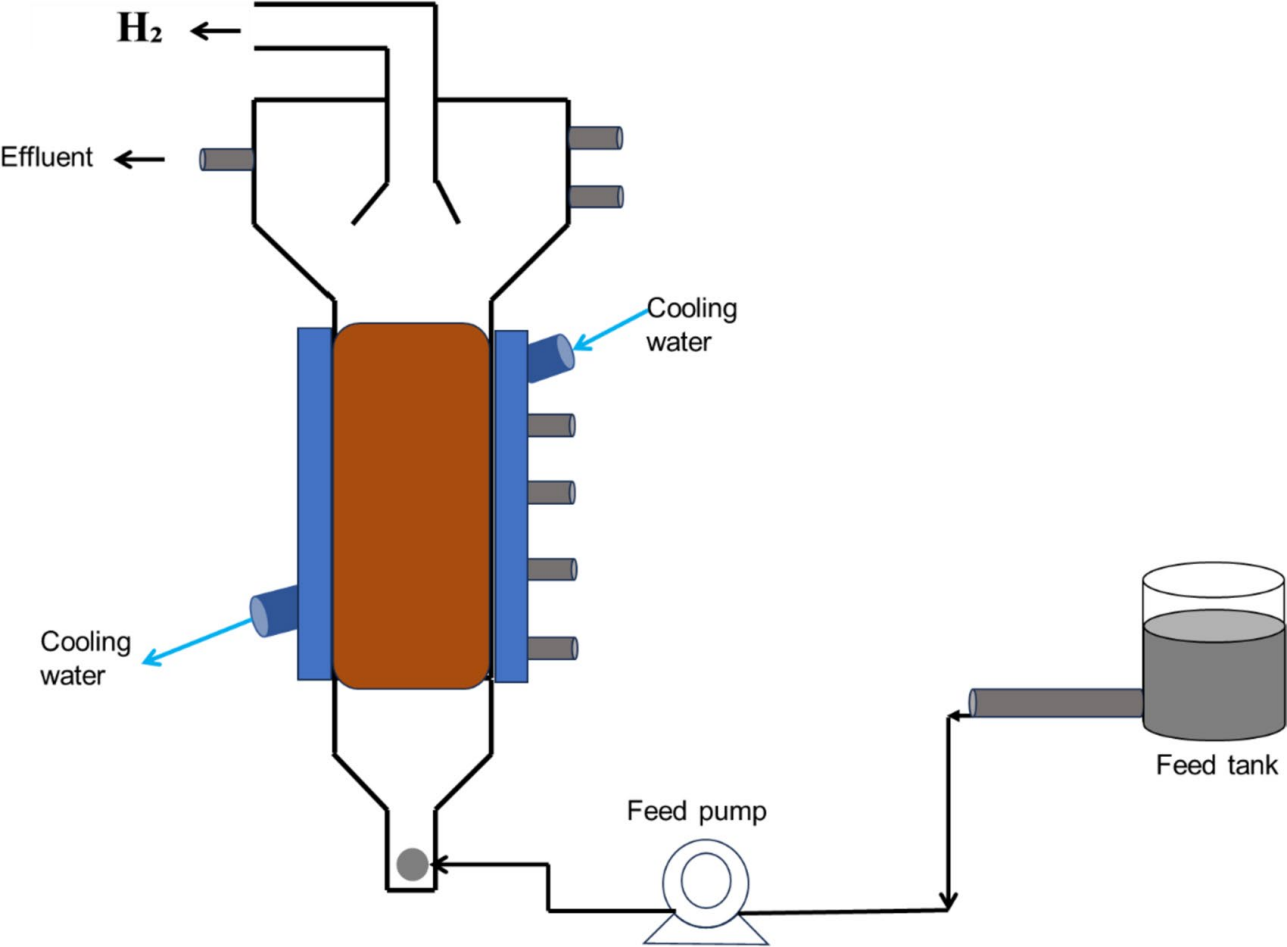
Upflow anaerobic sludge blanket (UASB) bioreactor modified from (Park et al. 2021)

UASB configurations (see Fig. 6) have been used for biohydrogen production during dark fermentation and operating on a spontaneous granulation (where the biomass accumulates in flocs) leading to high biomass concentrations. Based on productivity, this reactor configuration has demonstrated high biohydrogen production rates and yields as reported in several reviews (Kisielewska et al. 2015; Buitrón et al. 2019; Cruz-López et al. 2022). Immobilization techniques have been applied therein with even better performance when compared to traditional CSTR configurations (Keskin et al. 2012). This was attributed to the limited cell washout in the USAB configuration as opposed to the CSTR configuration with planktonic cells, making the column a more robust configuration in terms of mechanical stability. However, the scalability potential of the UASB bioreactor is still to be fully demonstrated, as most studies are focused on laboratory-scale demonstrations, even though hydrodynamic studies have been conducted to understand the flow patterns and biohydrogen relationship of this configuration (Wang et al. 2010a). Nonetheless, since similar technologies have been utilized in wastewater treatment (without hydrogen production), that literature may inform the development and scale up of UASBs for biohydrogen production.

#### Column bioreactors (vertical tubular)

**Fig. 7:**
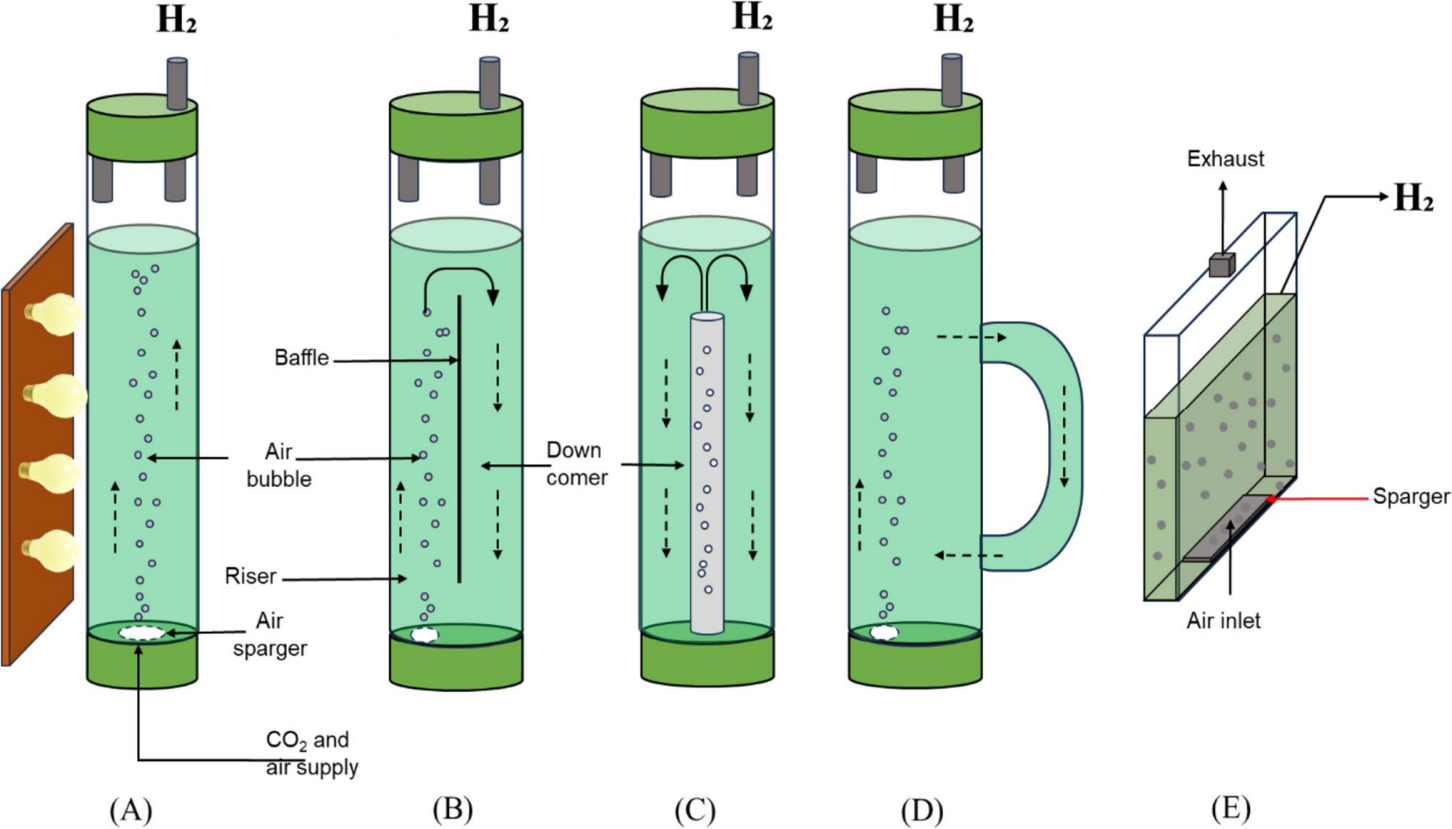
Column Bioreactors schematics: **A** Bubble column, **B** internal loop airlift, **C** Airlift with a concentric internal loop, **D** external loop airlift and **E** flat plate modified from (Gupta et al. 2015)

Column bioreactors have been demonstrated extensively in the literature for biohydrogen production. Based on the gas–liquid flow patterns, column bioreactors can feature airlift (Nayak et al. 2014; Zarei et al. 2021) or bubble column (Mirón et al. 2000; Carvalho et al. 2006) configurations (if gas is passed through the reactor) as shown in Fig. 7. The additions and modifications to a simple column volume are often done to improve the system hydrodynamics, resulting in better mixing and mass transfer than the conventional configuration. For instance, the airlift configuration (Fig. 7B) differentiates itself from the bubble columns (Fig. 7A) with the addition of a dividing wall baffle. In terms of scalability, these configurations have been demonstrated at scale, but not usually with the inclusion of light (for photofermentation)—that application still calls for further work at scale.

Another configuration used during photosynthetic biohydrogen production is the flat plate photobioreactors. This configuration has a simple geometrical structure (see Fig. 7E) based around its high surface area to volume ratio. Unlike bubble column and airlift bioreactor that hold specific orientation, flat plates do present different orientations (vertical, tilted, rocking motion, V-shaped and so forth) with increasing yield and productivity, as reported in several studies (Dasgupta et al. 2010; Benner et al. 2022; Sirohi et al. 2022). In terms of their scalability potential, flat plate reactors are likely to be scaled in a modular fashion rather than as a single larger vessel.

**Fig. 8:**
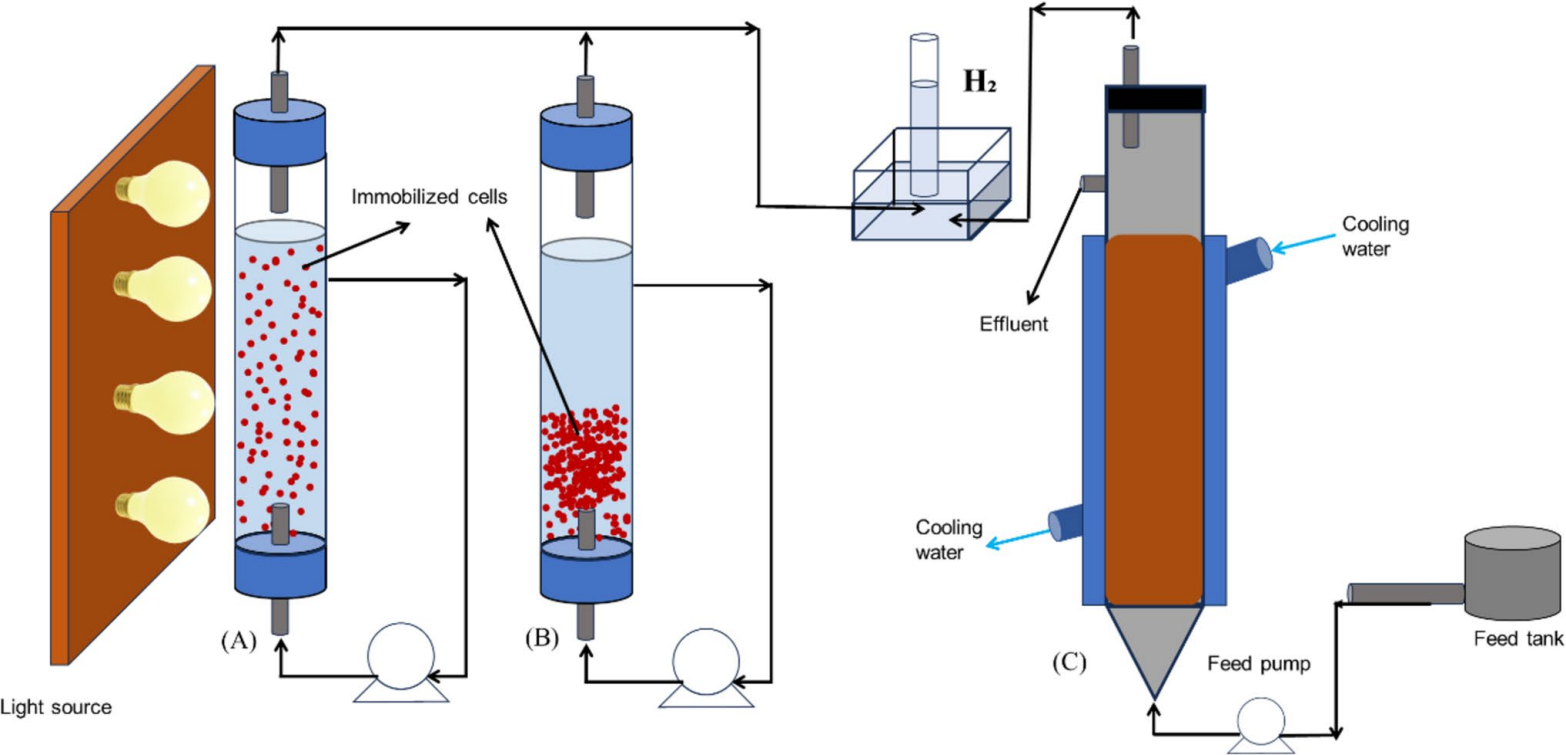
Schematic of a **A** Packed bed column, and **B** fluidized bed column, and **C** Fixed bed column modified from (Anzola-rojas et al. 2016; Kongjan et al. 2018; Ross and Pott 2020)

Aside from bubble column, airlift, and flat plate bioreactors, other types of vertical tubular bioreactors have been demonstrated for the production of biohydrogen, using the transport of media for mixing rather than gas sparging. Some examples include packed bed, fluidized bed, and fixed bed bioreactors, as shown in Fig. 8 (Zhao et al. 2019; Ross and Pott 2020). The packed and fluidized bed do operate with immobilized cells which builds on their dynamic for higher homogeneity while opening its scope for scalability. The fixed bed operates based on a support matrix, all of which can be comparatively expensive. However, comparatively high biohydrogen production has been recorded in these different tubular bioreactors while building an advancement of knowledge on how to separate hydraulic and solids retention time which is a growing area of interest in biohydrogen production (Sivagurunathan et al. 2016a; Ross and Pott 2020).

So far, these configurations have been applied mostly on laboratory-scale, shown between 250–5000 mL (Nayak et al. 2014; De Vree et al. 2015; Ross and Pott 2020; Zarei et al. 2021; Andreani et al. 2022; Mikheeva et al. 2022) and industrial applicability will require upscaling investigations. Regarding scale-up, the height and diameter of the column are of essence to control the bubble distribution and flowrate parameters—column pressure also is known to affect bubble expansion thus influencing bubble shapes and size (Kantarci et al. 2005; Shaikh and Al-Dahhan 2013). Further, in the case of photosynthetic systems, volume scales faster than surface area, and so larger reactors will receive relatively less light, reducing efficacy.

#### Horizontal bioreactor

**Fig. 9:**
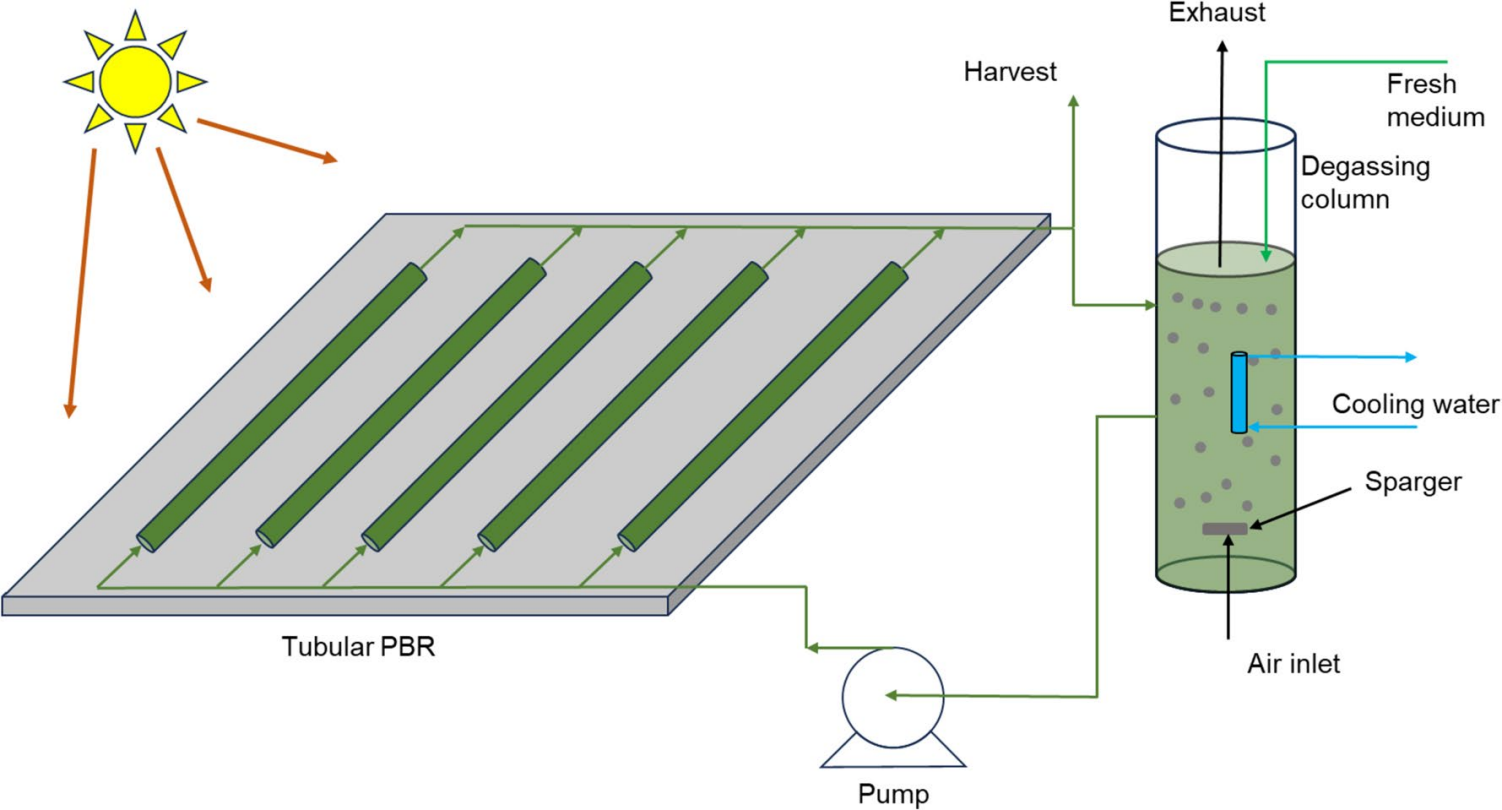
Horizontal tubular bioreactor. This is illustrated for outdoor applications featuring natural sunlight as the light source, although artificial light may also be utilized, modified from (Gebicki et al. 2010; Chanquia et al. 2022)

Horizontal tubular bioreactors are well suited for outdoor mass cultivation of biomass for photofermentative biohydrogen production, as demonstrated in Legrand et al. (2021). Most tubular outdoor bioreactors for biohydrogen production are glass or PVC or plastic tubes for good light penetration (Gebicki et al. 2010; Androga et al. 2011; Avcioglu et al. 2011; Adessi et al. 2012). So far, the ranges of used tube diameters are 40–120 mm which has shown to be a good surface-to-volume ratio for light penetration into the culture as seen with the illuminated surface areas from 1–2 m^2^ (Boran et al. 2010; Gebicki et al. 2010; Androga et al. 2011; Avcioglu et al. 2011; Adessi et al. 2012). Thus, this permits circulation of large volumes of microbial culture with the help of an external pump (Policastro et al. 2022). An example of this horizontal bioreactor configuration is shown in the Fig. 9.

In terms of scalability, this configuration has seen large-scale applications ranging from 1 to 90 L of producing volume (Sivagurunathan et al. 2016a; Policastro et al. 2022), and even more reported by Legrand et al. (2021). However, several challenges come with the outdoor cultivation strategy employed in these bioreactors. In particular, outdoor conditions are particularly variable—with light intensity throughout the day, to zero at night, and temperatures varying significantly depending on location. Further, if a continuous flow of wastewater is being used to produce hydrogen, then the issue of contamination can become a limiting factor. Nonetheless, outdoor tubular reactors can be employed to good effect, particularly with photosynthetic systems, which may be applied to biohydrogen production.

#### Hybrid bioreactors

Apart from the above-mentioned bioreactor configurations for biohydrogen production, there are of course additional modifications and other configurations adapted to serve the same purpose. These adapted configurations (such as including a membrane or making a multistage bioreactors) are discussed below.

##### Membrane bioreactors

**Fig. 10:**
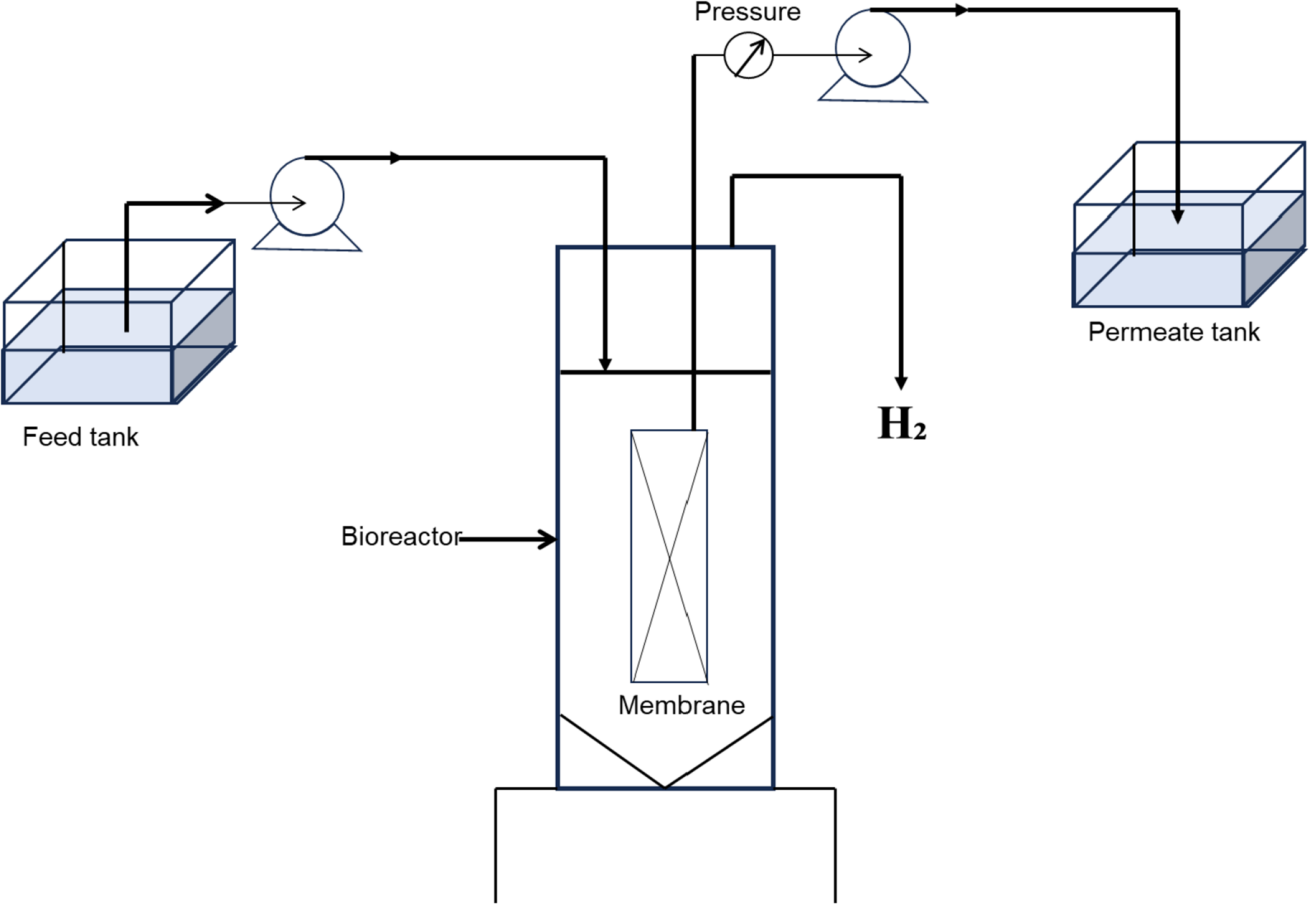
Hybrid membrane bioreactor with submerged membrane, modified from (Buitrón et al. 2019)

For membrane systems, the production of biohydrogen is done by incorporating a membrane to prevent solids flowing through (i.e. to retain the biomass within the reactor); the membrane can be positioned either externally or internally. For internal configurations, in the literature UASB or tubular bioreactors have been retrofitted with ultrafiltration membranes into their traditional configurations or from a side reactor in the traditional configuration as seen in Fig. 10. This adaptation increases productivity but membrane fouling is still a pertinent issue to be addressed (Aslam and Kim 2019; Buitrón et al. 2019).

**Fig. 11:**
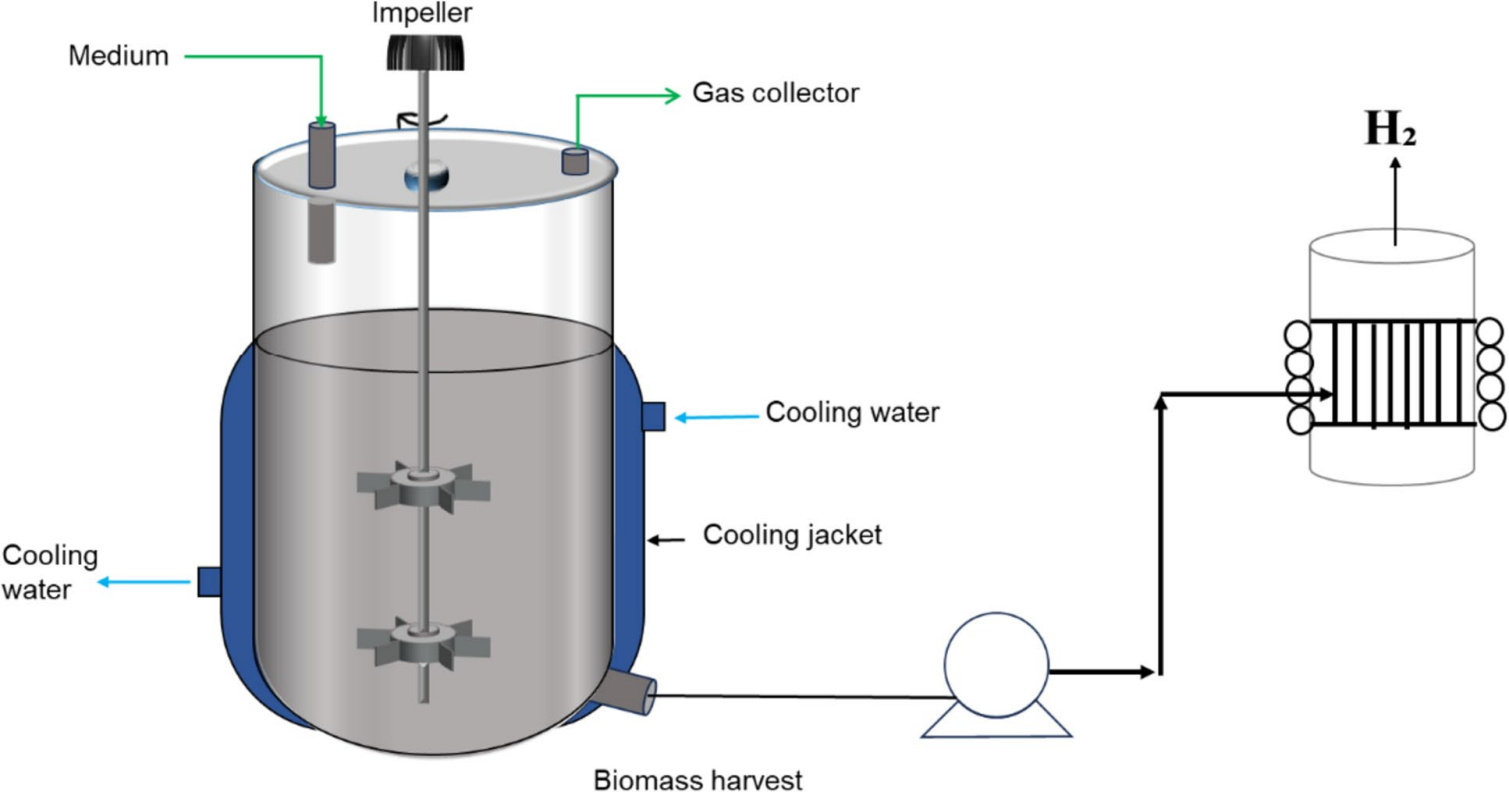
Hybrid membrane bioreactor with external membrane, modified from (Bakonyi et al. 2017)

For externally fitted configurations, some examples have demonstrated CSTR configurations that have been retrofitted with a non-porous polydimethylsiloxane membrane as shown in Fig. 11 (Bakonyi et al. 2017). These modifications lead to increased biohydrogen production rates to 8.9–9.2 L H_2_/L-day from 6.96–7.35 L H_2_/L-day when just a CSTR configuration was used. Such changes were driven by sparging the bioreactor with concentrated carbon-dioxide fraction from the membrane unit that likely attributed to a better mass transfer assisting to sweep hydrogen out to the CSTR headspace. Although membrane fouling of such systems was seen to be limited, one bottleneck arises from the decreased exchange area. Thereby necessitating the need to pump the medium through the external loop from the bioreactor leading to an increased energy demand. However, the major advantage of this system as compared to the internal loop is the ease in cleaning and eventually replacement of the membrane.

In terms of scalability, this type of membrane system is still reliant on a parent configuration for the exchange of fluid, and comparatively little work has been done on scale-up to date. Hence, more studies are required if these adaptations are to be used industrially. Additionally, the cost of membranes and the added complexity of the reactor system remain factors that may be important to investigate.

##### Multistage bioreactors

**Fig. 12:**
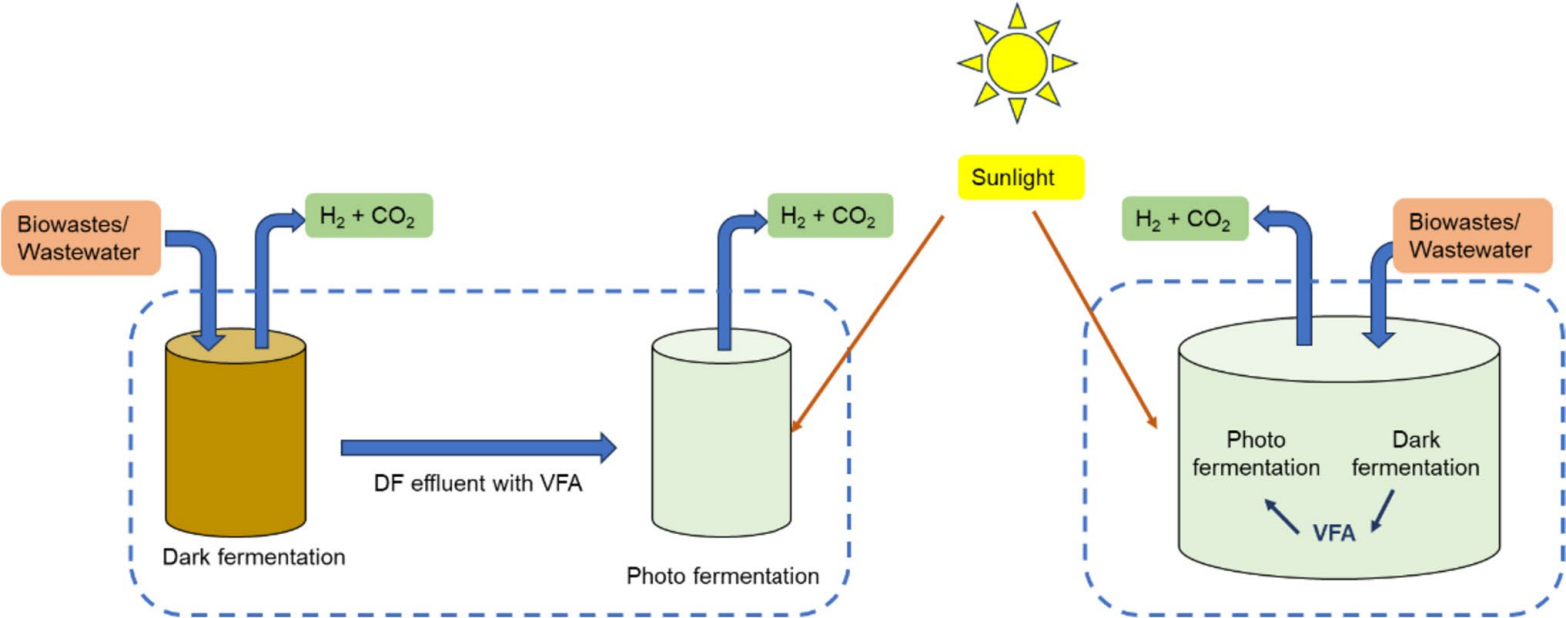
Schematic of multi-stage bioreactor **A** Sequential two-stage integrated DF and PF process and **B** Single-stage integrated DF and PF process, modified from (Cheng et al. 2022)

**Fig. 13:**
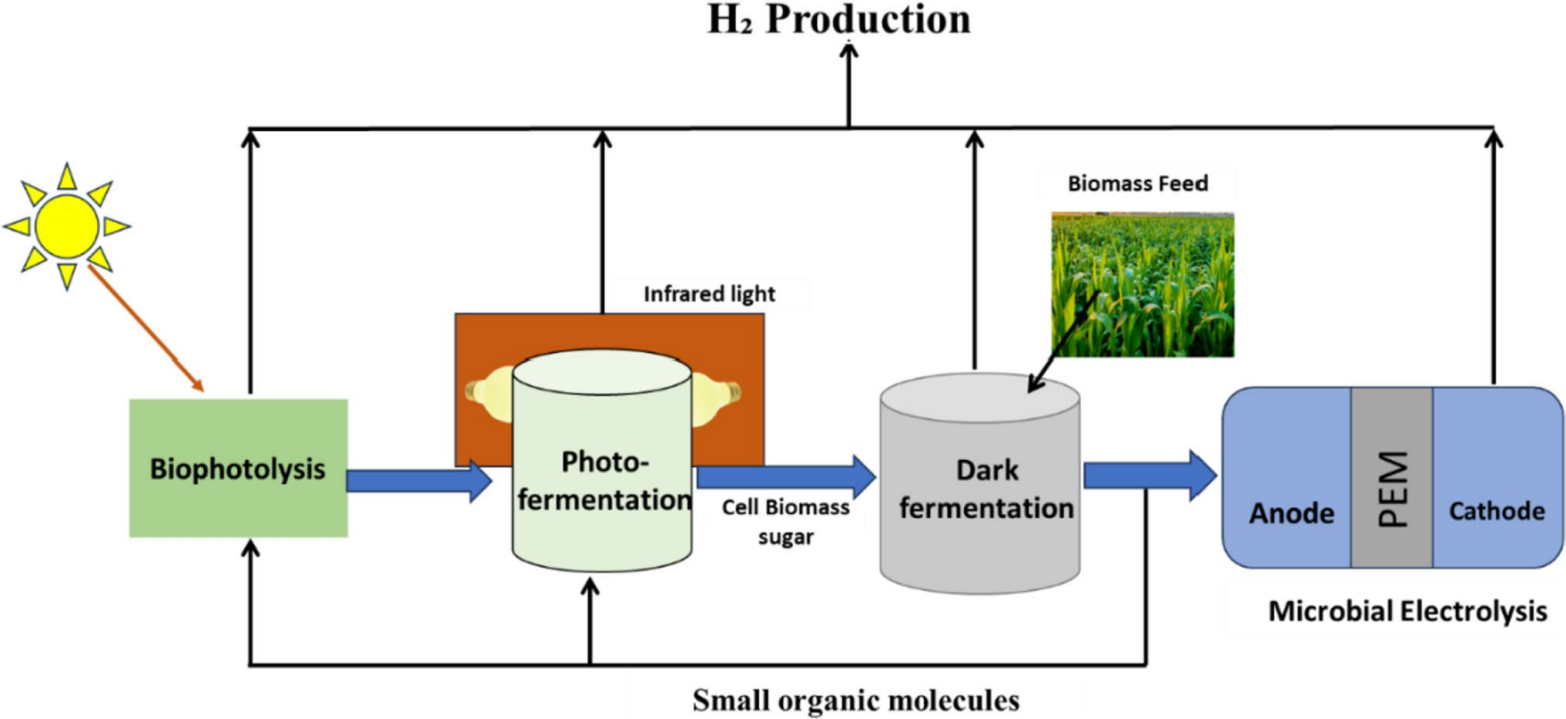
Schematic of multi-stage hydrogen production including biophotolysis, photo fermentation, dark fermentation, and microbial electrolysis, modified from (Department of Energy 2007)

A frequently suggested multistage system combines both dark- and photo-fermentation, as illustrated in Fig. 12. Indeed, more complex and highly integrated systems have been demonstrated, such as that shown in Fig. 13. In the first configuration a dark fermentation reactor takes biomass of some sort, anaerobically digests it to produce hydrogen and an organic acid rich wastewater, which is then fed to a photofermentation reactor where the organic acids are converted into hydrogen. This improves the overall conversion efficiency, although it does come at the cost of additional complexity.

The latter configuration was proposed with the idea of maximizing biohydrogen production and yield (Department of Energy 2007). In this process, the first reactor utilizes biophotolysis with algae or cyanobacteria, and the biomass feed is then transferred to the secondary reactor for dark fermentation. In the first reactor, two stages are combined i.e., photolysis and photo-fermentation using sunlight or artificial illumination to produce biohydrogen. In the secondary reactor, bacteria are used to produce biohydrogen and potentially organic acids from the biomass feedstock and transferred effluent (Department of Energy 2007; Singh et al. 2021). To reduce production costs, the organic acid produced from the secondary level could be transferred to the primary photo-fermentation stage. If this is not the case, the organic acid could be transferred to the fourth stage for the production of biohydrogen—in this stage microbial electrolysis cells will utilize the acid for biohydrogen production either at night or in light-limited conditions (Department of Energy 2007; Wang et al. 2011).

Although this system has merit, implementation is challenging as an in-depth understanding is required from bioreactor design to process controls and operation capabilities.

## Modelling of biohydrogen production systems

**Fig. 14:**
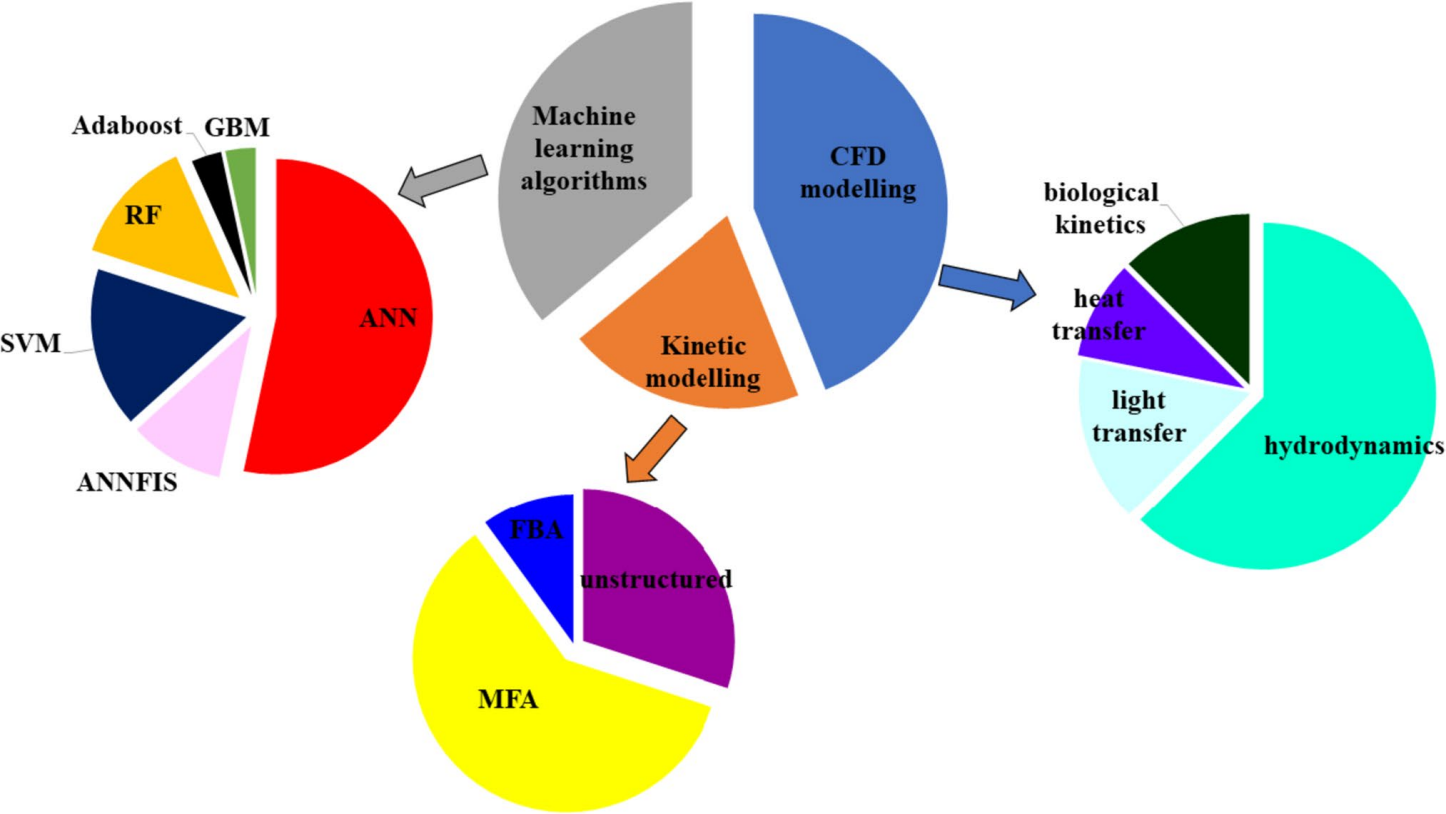
Summary of over 55 reviewed papers within mathematical modelling of biohydrogen production systems. The annotations are *CFD* Computational Fluid Dynamics, *FBA* Flux Balance Analysis, *MFA* Metabolic Flux Analysis, *ANN* Artificial Neural Networks, *ANNFIS* Artificial Neural Networks Fuzzy Inference Systems, *GBM* Gradient Boosting Machine Learning, *Adaboost* Adaptive Boosting Machine Learning, and *SVM* Support vector Machines

The rapid advancement in computer processors has enabled faster computation of mathematical problems previously deemed computationally infeasible. This has positioned mathematical modelling at the forefront of complementing experimental designs for the understanding, designing, optimizing, and upscaling of complex biological processes. The contributions of mathematical modelling towards accelerating biotechnological development, reducing the labor and expenses required for the optimal physical design and operating conditions are reviewed here, specifically with regards biohydrogen production. The significant amount of literature on this subject area can be grouped under four major methods, namely: (1) Kinetic (structured and unstructured) and constraint-based modelling, (2) Computational Fluid Dynamics (CFD) modelling, (3) Machine Learning, and (4) Hybrid modelling. Among these sections, the first three [i.e., (1) to (3)] have been comprehensively investigated in the literature as summarized in Fig. 14. However, the last method [i.e., (4)] is yet to be implemented in biohydrogen, to best of our knowledge, and thus is excluded from this review.

### Kinetic (structured and unstructured) and constraint-based modelling

Kinetic modelling aims to translate the biological processes of biomass growth, substrate consumption, and biohydrogen production into solvable mathematical equations. This modelling strategy is broadly grouped into structured and unstructured kinetic modelling. Structured modelling approaches physiologically describe the microbial regulatory adaptations to their micro-environment during biohydrogen production, based on intracellular metabolic reactions. Conversely, the micro and macro-environments are assumed to be confounded in the unstructured modelling approaches, hence are based on extracellular reactions. Of the two approaches, unstructured modelling for both photo and dark fermentation route of biohydrogen production have received significant attention over the years (i.e., 2010 to 2022) as comprehensively reviewed by these authors (Wang and Wan 2009; Nath and Das 2011; Chezeau and Vial 2019; Yahaya et al. 2022). The effects of culturing variables such as pH, temperature, dissolved substrate concentrations, dilution rates, light intensity and wavelength, light attenuation, hydraulic retention time (HRT), on the biohydrogen productivity have been discussed in detail by the authors (Wang and Wan 2009; Nath and Das 2011; Chezeau and Vial 2019; Yahaya et al. 2022), and thus omitted herein. To update these trends, the recent works by Anye Cho et al., (2021a, b) embedding light/dark cycles’ effects via $\eta$ and biohydrogen partial pressures via $\phi$ within photobioreactors of different configurations (i.e., Schott bottle PBR and Vertical tubular PBR) and scales (500 mL to 1 L) is presented in Eq. (1) and (2).By recalibrating $\eta$ and $\phi$, the biohydrogen productivity of a pilot scale plant can be infer from a bench top photobioreactor, thus accelerating the bioprocess development.

1 $$\frac{dX}{dt}={\mu }_{max}\cdot \mu (T)\cdot \left(\frac{\frac{{I}_{0}}{\left(\tau \cdot X\right)\cdot {L}_{L}}.(1-{e}^{-\left(\tau \cdot X\right)\cdot {L}_{L}})}{{k}_{s}\left(\frac{1}{\eta }\right)+\left(\frac{{I}_{0}}{\left(\tau \cdot X\right)\cdot {L}_{L}}.(1-{e}^{-\left(\tau \cdot X\right)\cdot {L}_{L}})\right)}\right)\cdot X$$

2 $$\frac{d{H}_{2}}{dt}=\phi \cdot {\alpha }_{{H}_{2}}(T)\cdot \left(\frac{\frac{{I}_{0}}{\left(\tau \cdot X\right)\cdot {L}_{L}}.(1-{e}^{-\left(\tau \cdot X\right)\cdot {L}_{L}})}{{k}_{s,{H}_{2}}\left(\frac{1}{\eta }\right)+\left(\frac{{I}_{0}}{\left(\tau \cdot X\right)\cdot {L}_{L}}.(1-{e}^{-\left(\tau \cdot X\right)\cdot {L}_{L}})\right)}\right)\cdot X$$

where $X$ is the biomass concentration (g L^−1^), ${H}_{2}$ is biohydrogen production (mL), ${\mu }_{max}$ is the maximum specific growth rate (h^−1^), $\phi$ biohydrogen enhancement coefficient, $\mu \left(T\right)$ and ${\alpha }_{{H}_{2}}(T)$ denote the respective effects of temperature ($T$) on biomass growth and biohydrogen production, ${k}_{s}$ and ${k}_{s,{H}_{2}}$ are light saturation coefficients (Wm^−2^) for biomass growth and biohydrogen production, respectively, $\eta$ is the effective light intensity coefficient, ${I}_{0}$ is the incident light intensity (Wm^−2^), $\tau$ (m^2^ g^−1^) is the light absorption coefficient, and ${L}_{L}$ (m) is the light path length.

On the other hand, structured modelling approaches are rarely investigated in the biohydrogen production literature. A key challenge being the unavailability of experimental measurements for the thousands of intracellular metabolites required for identification of fully formulated kinetic model parameters describing the metabolite transformations. Instead, constraint-based modelling approaches capable of describing the metabolite transformations without detail kinetic expressions are often sort after. Reviewing this approach showed two employed techniques, namely (1) Flux Balance Analysis (FBA) (see Eqs. 2.1, 2.1, 2.3) and (2) Metabolic Flux Analysis (MFA) (see Eqs. 3.1, 3.2, 3.3, 3.4). Both techniques aim to optimize a biological objective (e.g., maximize biohydrogen flux,${v}_{{H}_{2}}$ in Eq. 2.1), subject to state constraints (e.g., steady state in Eq. 2.2 and Eq. 3.2), reaction and flux constraints (lower and upper bounds in Eq. 2.3 and Eq. 3.4). Among the two techniques, MFA was observed to be more commonly utilized compared to FBA in the literature, as summarized in Fig. 14. This is due to the undetermined nature of FBA with more unknown fluxes than equations to be solved, thus leading to a plethora of possible solutions. For instance, the authors (Kaushal et al. 2018) had a stoichiometric matrix of 152 $\times$ 206 (i.e., number of metabolites by number of metabolic reactions), thus 54 degrees of freedom. Hence, their model returned flux distributions and not the unique fluxes when the FBA’s macroscopic environments of sole glucose, sole glycerol, and glucose-glycerol mixture were varied for the linear optimization of model [i.e., Eqs. (2.1, 2.1, 2.3)]. Unlike the large stoichiometric matrix in FBA, MFA utilized experimentally measured metabolite concentrations and estimated rates (i.e., fluxes) to further constrain the flux solution space, thus reducing the stoichiometric matrix size and employing the least square algorithm for optimization of model Eqs. (3.1, 3.2, 3.3, 3.4). For instance, there were 30 metabolites and 6 degrees of freedom in the glucose metabolism of *Klebsiella pneumoniae* ECU-15. The model identified macro-environmental conditions (i.e., initial glucose above 5 g/L and pH 7.0–7.5) enhancing hydrogen flux (Niu et al. 2011). Also, modelling for mixture culture of *Lactobacillus, Lachnospiraceae, Enterococcae*, *Clostridium* and *Bifidobacterium genera*, the authors (Gonzalez-Garcia et al. 2017) utilized 45 metabolite and 38 metabolic reaction and reported the low biohydrogen yield to be associated with by-product production and high fluxes through tricarboxylic acid (TCA) cycle and glycolysis (Gonzalez-Garcia et al. 2017). For the glycerol metabolism of *Clostridium tyrobutyricum*, the authors (Cheng et al. 2013) utilized 16 metabolites and 21 metabolic reactions, and explained the improved hydrogen production due to increased HRT via the flux for lactate, butyrate, and acetate (Cheng et al. 2013). Similar modelling of glycerol metabolism for *Clostridium Pasteurianum* by the authors (Sarma et al. 2017) employed 21 metabolites and 26 metabolic reactions, and showed enhanced flux towards butyrate under sonication leading to greater hydrogen production.

2.1 $$\mathrm{max} {w}^{T}\cdot {v}_{{H}_{2}}$$

2.2 $$s.t. \mathrm{S}\cdot \mathrm{v}=0$$

2.3 $${v}_{LB}\le v\le {v}_{UB}$$

3.1 $$\mathrm{min} \sum_{i=1}^{k}{\left({r}_{i}-{r}_{i,m}\right)}^{2}$$

3.2 $$s.t. \mathrm{S}\cdot \mathrm{v}=0$$

3.3 $$\mathrm{R}\cdot \mathrm{v}=\mathrm{r}$$

3.4 $${v}_{LB}\le v\le {v}_{UB}$$

where ${w}^{T}$ is a vector of weights, ${v}_{{H}_{2}}$ is biohydrogen flux, $\mathrm{S}$ is stoichiometry matrix with row entry for metabolites and columns entry for metabolite reactions, $\mathrm{v}$ is column vector of metabolite fluxes, ${v}_{LB}$ and ${v}_{UB}$ corresponds to the respective lower and upper bounds to the metabolite fluxes, $\mathrm{R}$ is the measurement matrix, ${r}_{i}$ and ${r}_{i,m}$ are the externally predicted and measured metabolite rates.

Although MFA proved to be invaluable for understanding the factors influencing hydrogen flux, the full potential of MFA for metabolic engineering of microbial cells was not fully unleashed in these studies. For example, the literature algorithms such as OptKnock (Burgard et al. 2003), Reacknock (Xu et al. 2013) and MIQP (Gerken-Starepravo et al. 2022) using binary variables to mathematically activate and/or deactivate fluxes could be embedded within these MFAs to maximize the biohydrogen flux. Using the work of Gonzalez-Garcia et al. (2017), such algorithms can knock out the unnecessary fluxes channeling to the by-products and through TCA and glycolysis, thus redirecting fluxes towards biohydrogen production. Therefore, it would be interesting to compare the biohydrogen productivity from such a computationally designed strain vs the wild-type microbial strain in future studies.

### Computational fluid dynamics (CFD) modelling

**Fig. 15:**
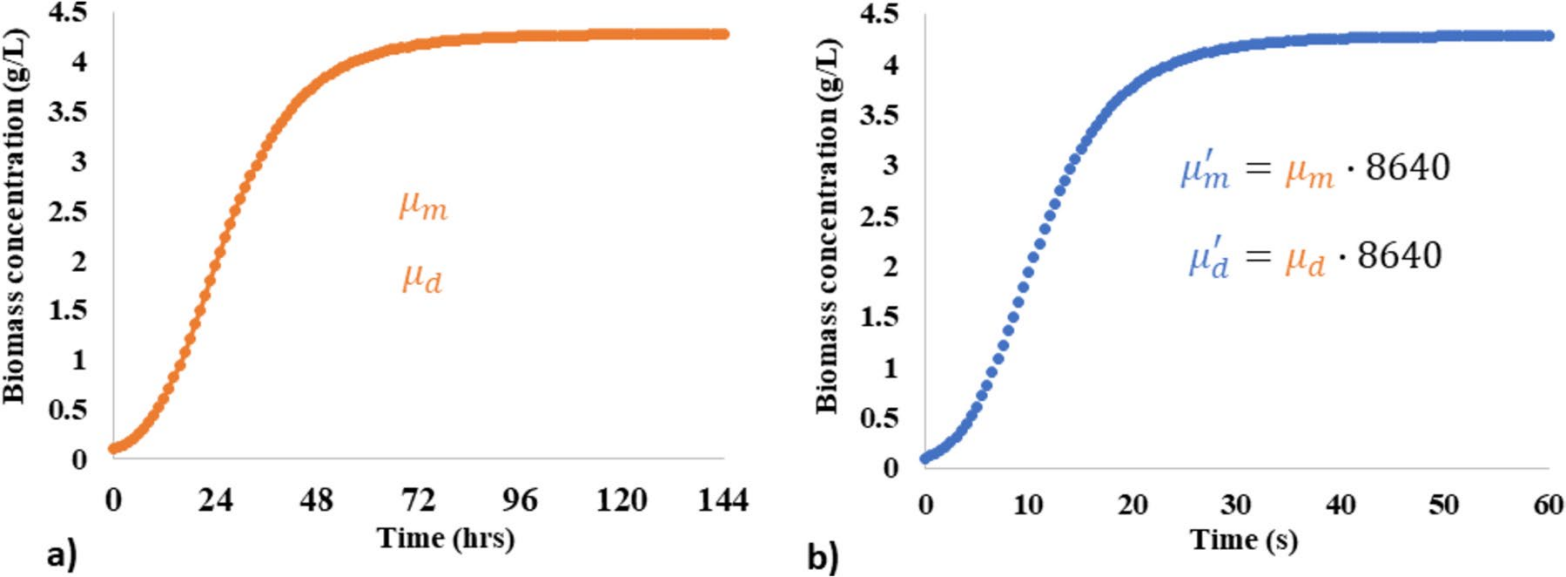
The accelerated growth kinetic approach by Anye Cho et al., (Anye Cho et al. 2021a): **a** 6 days (~ 144 h) fermentation simulation scaled to, **b** 60 s (~ 1 min) CFD simulation, without compromising the sigmoidal growth profile, gotten from (Anye Cho et al. 2023)

The complex bioconversions within bioreactors to produce biohydrogen can be decoupled into three or four subsystems, namely: (1) hydrodynamic mixing, (2) heat and/or mass transfer, (3) bioreactions transport, and (4) light transmission for the mathematical modelling of any one subsystem or all. CFD is a discipline exploiting numerical solvers to visualize these subsystems, thus accelerating biotechnological development and optimization by reducing the required labor and expenses for optimal physical designs. Table 3 summarizes the literature reported CFD investigations over the last decade modelling bioreactors from benchtop scale (i.e., 0.126 L) to industrial scale (i.e., 140,000 L) with about 62% pertaining to hydrodynamic mixing. This was not surprising as hydrodynamic mixing alleviates nutrient gradients and microbial cell sedimentation. Therefore, hydrodynamic mixing brings the microbial cells in contact with growth nutrients for bioconversions and alternates the microbial cells between the light and dark zones for photofermentation routes in PBRs, thus enhancing the biohydrogen productivity. So, the bioreactor parameters like reactor geometry (Wu 2013; Bosman et al. 2023a; Zhang et al. 2023b), impeller configurations (Ding et al. 2010; Wang et al. 2010b; Niño-Navarro et al. 2016; Maluta et al. 2019), mixing speed (Ding et al. 2010; Srirugsa et al. 2017, 2019; Brindhadevi et al. 2021; Jabbari et al. 2021) and HRT (Wang et al. 2009; Wu 2013; Srirugsa et al. 2019; Brindhadevi et al. 2021) were investigated with CFD towards enhancing hydrodynamic mixing. As well, only a handful of these studies were based on novel bioreactor configurations as summarized in Table 3. Meanwhile most were directed towards improving biohydrogen productivity in the traditional continuous stirrer tank (CSTR) designs. This retro-fitting strategy with optimized CSTR-impellers enabled faster bioprocess development and biotechnology transfer to industrial scale as seen in the works of Wang et al. 2010b successfully upscaling from 17 L to 140,000 L with CFD. Without experimental validations, the qualitative and quantitative CFD results cannot be verified. Therefore, these CFD studies were commonly validated by manufacturing the CFD suggested design and performed experimental quantification of the biohydrogen productivity. However, the hydrodynamic flow fields interact with the other subsystems [i.e., (2), (3), and (4)], but are partially or completely neglected in the aforementioned 62% CFD-based frameworks. Therefore, the validated designs are deemed suboptimal. Robustly, an optimal configuration should therefore integrate subsystems (1) to (3) for fermentation studies like (Wang et al. 2010a; Maluta et al. 2019; Jabbari et al. 2021; Wodołażski and Smoliński 2022; Zhang et al. 2023b) and (1) to (4) for photo-fermentation studies like (Anye Cho et al. 2023), into a whole system CFD-coupled biokinetic framework. However, the computational resource requirements for solving this type of whole system CFDs are enormously high [i.e., lasted a few months to convergence (Anye Cho et al. 2023)], thus presenting a bottleneck. Even in the event of powerful computer processors, and parallel computing, such CFD simulations are expected to take several months to simulate a few days of bioprocess fermentation, and the computational cost scales with the bioreactor size under simulation. On the other hand, numerical approximation techniques such as the accelerated growth kinetic strategy by Anye Cho et al. (2021a) have shown remarkable CFD simulation cost reductions. The approach solves the scalar transports equations, for instance, Eq. (4) for biomass (Anye Cho et al. 2021a), but similarly substrate consumption and biohydrogen productivity in the works of Anye Cho et al., (Anye Cho et al. 2023), by scaling the biokinetic parameters, ${\mu }_{m}$ and ${\mu }_{d}$, with a factor of 8640 to reduce the overall CFD simulation time by several order of magnitude without adversely affecting the fermentation growth curve as illustrated in Fig. 15. Therefore, the 144 h experimental cultivation time becomes representative of a 60 s CFD simulation with ${\mu }_{m}{\prime}$ and ${\mu }_{d}{\prime}$, providing CFD cost simulation savings from months to days.

4 $$\frac{dX}{dt}+\left[\nabla \cdot \left(\overline{{u }_{L}}X\right)\right]=\nabla \cdot \left(\left({D}_{X,L}+\frac{{ \mu }_{L,Turb}}{{{\rho }_{L}Sc}_{t}}\right)\nabla X\right)+\left({\mu }_{m}{\prime}\cdot \frac{{I}_{z}}{{k}_{s}+{I}_{z}+\frac{{I}_{z}}{{k}_{i}}}\cdot X-{\mu }_{d}{\prime}{\cdot X}^{2}\right)$$

where the first and second terms on the left-hand side of Eq. (4) denote the accumulation and convection of the biomass,$X$, meanwhile those of the right-hand side denote the biomass diffusion and growth or decay, respectively. $\overline{{u }_{L}}$ is Reynolds average liquid phase velocity, ${\rho }_{L}$,${D}_{X,L}$, ${Sc}_{t}$ and ${\mu }_{L,Turb}$ are the liquid phase density (comparable to biomass density), biomass viscosity, turbulent Schmidt number, and effective viscosity respectively. ${\mu }_{m}{\prime}$ and ${\mu }_{d}{\prime}$ are accelerated growth and decay kinetic parameters, ${I}_{z}$ is the local light intensity, ${k}_{s}$ and ${k}_{i}$ are light saturation and light inhibition coefficients, respectively.

**Table 3:** Literature investigation of biohydrogen productions in bioreactors via CFD simulations of hydrodynamics, biological kinetics, light and heat transport

CFD coupled sub models	Bioreactor type	Scale	References	Remarks
Hydrodynamics, biological kinetics, light transfer	Vertical tubular	1 L	Anye Cho et al. (2023)	Simulation cost savings enabling the estimation of CFD uncertainty via Monte Carlo simulation for the first time
Hydrodynamics	Continuous stirrer tank (CSTR)	5 L	Srirugsa et al. (2017)	Obtained highest biohydrogen yield of 325 mL H_2_ gCOD^−1^ at a mixing speed of 150 rpm
Hydrodynamics, biological kinetics	Column	0.2 L	Zhang et al. (2023b)	Vertical filling strategy showed more evenly distributed velocity magnitude than horizonal, maximum biohydrogen yield of 2.6 mol mol^−1^ glucose
Hydrodynamics, biological kinetics	Expanded granular sludge bed (EGSB) reactor	3.35 L	Wang et al. (2010a)	Demonstrated qualitative relationships between hydrodynamics and biohydrogen production
Light transfer	Thermosiphon photobioreactor	1 L	Bosman et al. (2023a)	Increased biohydrogen production by 48% with addition of light reflector systems
Review of the sub model coupling strategies	Anaerobic lagoon, plug-flow digester, complete-mix digester, anaerobic biohydrogen fermenter, anaerobic biofilm reactor, and photobioreactor	All scales	Wu (2013)	Reviewed 6 types of bioreactors and the issues of simulating rheology biomaterials were addressed
Hydrodynamics	Plexiglass CSTR	19.6 L	Montante et al. (2013)	Novel CSTR with external gas recirculation towards a gas separator for biohydrogen recovery and purification without additional energy
Hydrodynamics	EGSB	3.35 L	Wang et al. (2009)	Demonstrated the controlling of HRT to be a critical factor in biohydrogen production
Hydrodynamics	CSTR	17 L to 140,000 L	Wang et al. (2010b)	Optimized the impellers for velocity magnitude and stagnation zones
Hydrodynamics heat transfer	Annular	1 L	Basak et al. (2016)	Three factor optimization of malic acid, glutamic acid, and FeCl_3_ with response surface methodology (RSM) and achieve 7.0 mL H_2_ L^−1^h^−1^ biohydrogen productivity
Hydrodynamics	CSTR	25 L	Niño-Navarro et al. (2016)	Impact of impeller configuration on biohydrogen production, the optimum showed highest biohydrogen of 407.94 mL Lh^−1^
Hydrodynamics	Vertical and horizontal CSTR	18 L	Brindhadevi et al. (2021)	Effects of HRT, impeller speed, vortex growth, and pH on biohydrogen production
Hydrodynamics	CSTR	5 L	Srirugsa et al. (2019)	Effects of HRT, and mixing speeds (10, 50, 100 and 150 rpm) on biohydrogen production, maximum yield at 150 rpm equivalent to 6,958 mL per litter palm oil mill effluent
Hydrodynamics, biological kinetics	CSTR	5.8 L	Maluta et al. (2019)	Dual impellers CSTR and detail estimation of different relevant variables and interaction on a local basis
Review paper: hydrodynamics, biological kinetics	Anaerobic digester (AD)	Local and global scales	Trad et al. (2016)	Reviewed mixing in fermentation and scale-up procedures
Hydrodynamics,	CSTR	17 L	Ding et al. (2010)	Different impeller configurations and speeds (50 to 70 rev/m) generated different flow patterns, and hence different efficiencies for biohydrogen production
Hydrodynamics, biological kinetics	Batch CSTR integrated Pd–Ag membrane Unit	5 L	Jabbari et al. (2021)	CFD-RSM optimized pH of 6.2, impeller speed of 115 and gas inlet rate of 2.4 $\times$ 10^–5^ producing maximum biohydrogen of 24.09 L
Heat transfer	Up-flow tubular bioreactor	126 mL	Zhiping et al. (2017)	Showed tiny temperature fluctuations, and the proposed reactor is suitable for photo-fermentation biohydrogen production
Review paper: hydrodynamics, biological kinetics	AD bioreactors	All scales	Saini et al. (2021)	Identified novel areas for AD technology advancement for renewable energy productions
Hydrodynamics, heat transfer	Up-flow baffle photo-bioreactor (UBPB)	2.7 L	Zhang et al. (2014a)	The inlet velocity markedly influenced the UBPB’s temperature distribution, optimized parameters yielded about 240.0 mmol L^−1^ cumulative hydrogen
Hydrodynamics, heat transfer	Annular triple jacketed PBR	1 L	Basak et al. (2022)	Optimized parameters showed agreeable simulated fittings with modified Gompertz equation versus the experimentally measured biohydrogen
Hydrodynamics, biological kinetics	Packed bed column	3.14 L	Wodołażski and Smoliński (2022)	Study showed metabolite (VFAs: acetic, propionic, butyric) constitute important parameters influencing biohydrogen production efficiency

The simulation cost reduction with this approach (Anye Cho et al. 2023) is enormous (i.e., from several weeks to a few hours), even enabling the estimation of bioprocess uncertainties within the CFD-coupled biokinetic framework for the first time, thus presenting a way forward for bioreactor optimization under uncertainty in future studies.

### Machine learning (ML)

So far, the above reviewed mathematical models have been first-principle-based and are generally time-consuming to develop as well as being highly complex, nonlinear, and stochastic to solve. For example, the first principle anaerobic digestion model no.1 (ADM1) consists of over 20 biochemical reactions and more than 30 biokinetic parameters to be optimized, and is thus analytically challenging to estimate all model parameters. Conversely, machine learning (ML) presents a subclass of artificial intelligences capable of modelling these complex biochemical reactions via knowledge discovery without the help of first-principle models. However, ML requires large datasets for training and knowledge discovery which are often difficult and/or expensive to generate experimentally. In the case of dataset availability, the ease of training ML (e.g., back propagation) favors their application for biological hydrogen production.

The conducted literature survey, as summarized in Table 4, reveals 6 supervised algorithms, namely: (1) Artificial Neural Network (ANN), (2) Artificial Neural Networks Inference Fuzzy Systems (ANNFIS), (3) Support Vector Machine (SVM), (4) Gradient Boosting Machine learning (GBM), (5) Random Forest (RF), and (6) Adaboost, with ANN dominating the investigations. As these algorithms have already been thoroughly elucidated elsewhere by Mowbray et al. (2021) Pandey et al. (2023), Sharma et al. (2022), thus omitted in this review. Instead, emphasis was placed on their applications pertaining to the modelling and/or optimization of biological hydrogen production. Generally, the reported models were either time invariant/independent (steady state models) or time variant/dependent (dynamic models). For the steady state models, fermentation time was not included as an input parameter, thus they predicted the biohydrogen yield (Sydney et al. 2020; Liu et al. 2020) biohydrogen productivity (Wang et al. 2021; Hosseinzadeh et al. 2022) at the final fermentation time step. Thereafter, the models were utilized for optimization and compared with Response Surface Methodology (Yadav et al. 2021; Wang et al. 2021; Liu et al. 2021; Nassef et al. 2022) as summarized in Table 4.

**Table 4:** Literature ML algorithms applied for the modelling and optimization of biohydrogen production processes

Authors	Process variables	ML algorithm(s)	Remarks
Monroy et al. (2018)	Light intensity, pH, and metal concentration (Fe, V, and Mo)	ANN	6–9-1 architecture, R^2^ 0.939, poor prediction for photobiological hydrogen production in the lag phase
Hosseinzadeh et al. (2022)	Wastewater concentrations of Acetate, butyrate, acetate/butyrate, ethanol Fe and Ni	GBM, SVM, RF and AdaBoost	Time invariant (i.e., final value) biohydrogen prediction with R^2^ 0.893, 0.885, 0.902 and 0.889 respectively
Taheri et al. (2021)	Organic loading, effluent, mixed liquor suspended solid and mixed liquor volatile suspended solids	ANFIS and ANN	Prediction of transmembrane pressure with R^2^ of 0.93 and 0.88 respectively. ANFIS outperform ANN
Nasr et al. (2013)	pH, substrate concentration, biomass concentration, temperature, and volumetric hydrogen production	ANN	Dynamic prediction of biohydrogen production, 5–6-4–1 architecture, R^2^ of 0.989. Average prediction error of less than 10% for three different tested case studies
Aghbashlo et al. (2016a)	Culture agitation speed, syngas flow rate, process exergetic efficiency, rational exergetic efficiency and normalize exergy destructions	Fuzzy network	Optimal syngas flowrate of 13.68 mL/min, culture agitation speed of 348.62 rpm, exergetic efficiency of 16.46%, rational exergetic efficiency of 91.56% and normalize exergy destructions of 2.14
Sydney et al. (2020)	Fermentation time, sum of all VAFs, acetate, butyrate, lactate, propionate concentrations	ANN	Predicted biohydrogen yield with high accuracy (R^2^ > 0.987)
Yadav et al. (2021)	Headspace, butyric acid supplementation, and temperature	ANN-RSM	3–10-1 architecture, R2 0.999, optimization led to a 78% maximum substrate energy recovery
Wang et al. (2021)	Fermentation time, Nickel nanoparticles, pH, temperature, initial substrate concentration	ANN-RSM	Optimal biohydrogen prediction with 1.21 (mol/mol glucose) with less than 10% experimental uncertainties
Sultana et al. (2023)	Pretreatment length, WFO concentration, and sample pH	Hybrid Bayesian and support vector regression	High adjected R2 > 0.99 for biohydrogen and biomethane. Performance enhancement of 64.16% and 9.81%, respectively
Sharma et al. (2023)	Light illumination, nanoparticle toxicity, cost–benefit evaluations, and life cycle effects	Review of ML algorithms	Different pathways of biohydrogen production
Nassef et al. (2022)	Reformer temperature, stem-to-fuel ratio, and biodiesel-to-glycerol ratio	Fuzzy network	Optimization increased hydrogen production by 5.74% and 4.8% compared to the experimental and the RSM methodology, respectively
Aghbashlo et al. (2016b)	Culture agitation speed, syngas flow rate, process exergetic efficiency, rational exergetic efficiency and normalize exergy destructions	Fuzzy network-ANN	Optimal syngas flowrate of 13.35 mL/min, culture agitation speed of 383.34 rpm, process exergetic efficiency of 21.66%, rational exergetic efficiency of 85.65% and normalize exergy destructions of 1.56
Sharma et al. (2022)	Biochemical route, temperature, retention time, pressure, feedstock composition,	Review of ML algorithms	Techno-economic and scientific obstacle to ML applications in agriculture waste biomass-based biohydrogen production
Pandey et al. (2023)	Variety of organic waste and biomass feedstock	Review of ML algorithms	Contemporary research and perspectives on ML application in biohydrogen production technology
Liu et al. (2020)	Carbon sources (glucose and xylose), inhibitors (acetate, furfural and aromatic), hydrogen yield and hydrogen evolution rate	ANN	Manipulating ratio of glucose to acetate at an optimal range (circa. 14:1) improves biohydrogen yield and evolution rate regardless of microbial strains
Liu et al. (2021)	Chemical additions, especially nano-particles towards hydrogen yields	ANN-RSM	Optimal range of particle size, chemical addition, enhancing hydrogen yield and hydrogen evolution rates were identified

The abbreviations ANN, ANNFIS, SVM, GBM, and RF correspond to Artificial Neural Network, Artificial Neural Networks Inference Fuzzy Systems, Support Vector Machine, Gradient Boosting Machine learning, and Random Forest

Although satisfactory regression coefficients (i.e., R^2^ > 0.8) were observed in these studies, biological hydrogen production involves thousands of metabolite reactions that are typically time variants over the course of photo and/or dark fermentation. Hence, steady state models fall short of the underlining process description whereas dynamic models accounting for the process trajectory and the subsequent dynamic optimization are better suited. For instance, the authors, Monroy et al. (2018) constructed an ANN with 6-9-1 architecture having the photofermentation time as one of the ANN inputs. They reported satisfactory overall biohydrogen production prediction (i.e., R^2^ = 0.939) but poor prediction performance in the lag phase. Similar biohydrogen state prediction accuracies were observed in Nasr et al. (2013) and validated the complexities of the bioprocess dynamics requiring improved ML modelling approaches. One such example is the works by. Del Rio-Chanona et al. (2017) whereby the dynamic system’s rate of change at the next time step is estimated by feeding the ANN with essential bioprocess information at the current time step. Although yet to be applied for biohydrogen modelling, the strategy outperforms presented literature approaches (Nasr et al. 2013; Monroy et al. 2018) of feeding the ANN inputs with the current fermentation time to predict the next time step. Also, the Del Rio-Chanona et al. (2017) approach can predict multiple steps ahead when provided with only the initial conditions, thus minimizing prediction errors for the lag phase.

Whilst state-of-the-art ML applications for biohydrogen modelling have seen 6 main algorithms, a plethora of other algorithms are yet to be explored. For instance, Gaussian process models, requiring small training datasets and returning the mean prediction as well as the associated uncertainties are attractive for biohydrogen modelling and optimization. This is especially the case as the above-mentioned investigations did not consider bioprocess reliability under uncertainty. For instance, the bootstrapping technique can be utilized for estimating ANN prediction uncertainties.

## Techno-economics and life cycle assessment strategies for biohydrogen production

Currently, methane gas reforming, coal gasification, and electrolysis processes produce above 95% of the hydrogen currently in use (Ramprakash et al. 2022). Techno-economic analyses (TEA) and life-cycle analyses (LCA) of the complete hydrogen production value chain are crucial for commercializing hydrogen production using biological processes. TEA highlights the technical and economic feasibility of the process by analyzing the costs and revenues associated with the entire production process, including capital costs (CAPEX), operational costs (OPEX), feedstock costs, energy consumption, and product value. LCA highlights the environmental impact of the entire production process throughout the life cycle holistically, including greenhouse gas emissions, energy, water consumption, and waste generation. Hence, combining TEA and LCA for the biological hydrogen production process provides a comprehensive understanding of the technical, economic and environmental implications of biohydrogen commercialization. Limited studies are available in the literature covering the commercialization potential of biohydrogen both from a technical, economic and environmental perspective—most of the literature would carry out TEA without LCA; these are summarized in Table 5. This indicates that research needs to be done to realize the full commercialization potential of biohydrogen, focusing on the integrated TEA and LCA.

**Table 5:** Summary of biological-based hydrogen production costs

Feedstock	Synthesis route	Processing conditions and scale	Production cost ($/kg H_2_)	References
Microalgae	Direct photolysis	Large scale	0.53–13.53 $/kg H_2_	Amos (2004)
Potato peel starch (*agricultural waste*)	DF for H_2_ combined with AD for methane	Feed rate: 13.4 Tons/h Organisms: *Caldicellulosiruptor saccharolyticus* (DF) Hydrogen yield: 1.37–3.48 mol H_2_/ mole of glucose Large scale	7.20–48.96 $/kg H_2_	Ljunggren and Zacchi (2010)
Barley straw (*agricultural waste*)	DF combined with PF	Feed rate: 25 Tons/h Organisms: *Caldicellulosiruptor saccharolyticus* (DF) Organisms: *Rhodobacter capsulatus* (PF) Hydrogen yield: • 20 mmol H_2_/L/hr (DF) • 0.21 mmol H_2_/L/hr (PF) Large scale	60.77 $/kg H_2_	Ljunggren et al. (2011)
Wheat straw (*agricultural waste*)	DF for H_2_ combined with AD for methane	Feed rate: 2 Tons/h Organisms: *Caldicellulosiruptor saccharolyticus* (DF) Hydrogen productivity: • 2.8–6.1 L/L/d at composition of 46–57% H_2_, 43–54% CH_4_ and 0.4% CO_2_ Large scale	23.04 $/kg H_2_	Willquist et al. (2012)
Sugar beet molasses	DF combined with PF	Feed rate: 0.6–0.73 Tons/h Organisms: *Caldicellulosiruptor saccharolyticus* (DF) Organisms: *Rhodobacter capsulatus* (PF) Hydrogen productivity: • 16.3–50 mmol H_2_/L/hr (DF) • 0.5–3 mmol H_2_/L/hr (PF) Large scale	12.28–42.13 $/kg H_2_	Urbaniec and Grabarczyk (2014)
Algae	Direct photolysis Indirect photolysis PF DF	Feed rate: Glucose as substrate for DF and PF Theoretical study Large scale	1342 $/ kg H_2_ 2 $/ kg H_2_ 3.7 $/kg H_2_ 18.7 $/ kg H_2_	Sathyaprakasan and Kannan (2015)
Waste bread (Food waste)	DF	Feed rate: 2 Tons/day Organisms: From anaerobic sludge from local municipal waste Hydrogen yield: 15 mol H_2_/ mole of glucose Large scale	14.89 $/kg H_2_	Han et al. (2015, 2016a)
Food waste	DF	Feed rate: 10 Tons/day Organisms: *Biohydrogenbacterium* R3 (DF) Hydrogen yield: 1.52—2.13 mol H_2_/g glucose Large scale	25.73 $/kg H_2_	Han et al. (2016b, c)
Wood processing residues (*Pinus Patula*)	DF, compared to gasification and other biorefinery approaches including ethanol and electricity generation	Feed rate: ~ 14.1 Tons/h Organisms: *Thermoanaerobacterium thermosaccharolyticum* Hydrogen production: 0.068 Tons/h Large scale	Gasification (2.22–3.59 $/kg H_2_) 35.56 $/kg H_2_ 27.29 $/kg H_2_ when ethanol is produced as a co-product Environmental impact: Gasification: 0.30 kg CO_2_/ kg *Pinus Patula* DF -1.07 and -1.42 0.30 kg CO_2_/ kg *Pinus Patula* (without and with ethanol cascade respectively)	García et al. (2017)
Water + Algae	Direct photolysis	Large scale Theoretical study	2.57 $/ kg H_2_	Nikolaidis and Poullikkas (2017)
Renewable resource	Direct photolysis PF	Large scale Theoretical study	7.27 $/ kg H_2_ 7.61 $/ kg H_2_	Dincer and Acar (2015)
Food waste	DF	Feed rate: 100 Tons/day Organisms: *Thermoanaerobacterium thermosaccharolyticum* Hydrogen yield: 2.26 mol H_2_/mol-hexose Large scale:	3.2 $/ kg H_2_	Dinesh et al. (2018)
Agave bagasse (*agricultural waste*)	DF for H_2_ compared with AD for methane	Feed rate: batch systems, 73.64 g Organisms: Not disclosed for both DF and AD Hydrogen yield: 2.30–3.81 mol H_2_ / mol hexose Bench scale:	2.58 $/ L H_2_ 0.84 $/ L CH_4_	Tapia-Rodríguez et al. (2019)
Microalgae	Indirect photolysis	Capacity: 1200 TJ/annum Bench scale:	1.2 $/kg H_2_	Anwar et al. (2019)
Microalgae	Direct photolysis	Theoretical study Large Scale (review)	1.2 $/kg H_2_	Mona et al. (2020)
Wheat straw	DF	Feed rate: 100–2000 Tons/day Organisms: *Thermoanaerobacterium thermosaccharolyticum* Hydrogen yield: 406.98—445.51 kg-H_2_/h Large scale:	26.72–41.77 $/kg H_2_	Sanchez et al. (2020)
Brewery wastewater	DF	Feed rate: 400 million L/annum Organisms: *sewage sludge as inoculum* Hydrogen yield: 1.77 H_2_ mL/L/h Large scale:	7.35 $/kg H_2_	Mutsvene et al. (2023)
Molasses	PF	Feed rate: 20 L PBR Organisms: *Rhodobacter capsulatus YO3 (hup)*^*−*^ Hydrogen yield: 0.7 mmol H_2_/L/h Pilot scale:	1362 $/kg H_2_	Genç and Koku (2023)

A comprehensive theoretical study by Lee (2016) adopted a cost–benefit analysis approach to detail biohydrogen production's cost and revenue structure. It was estimated that biohydrogen would likely reach competitive production between the years 2008 to 2042, with production costs between 2–3 $/kg bioH_2._ These costs will be competitive with conventional fossil fuel-based production. Additionally, it was found that production costs are less sensitive to feedstock prices alone but rather more sensitive to the CAPEX and total OPEX (Lee 2015). Concerning CAPEX, minimizing costs associated with equipment and infrastructure for biological processes for hydrogen would be crucial as we move toward the commercialization of biohydrogen. Similarly, for OPEX, running expenses such as energy input and raw materials would need to be minimized for biological hydrogen production.

**Fig. 16:**
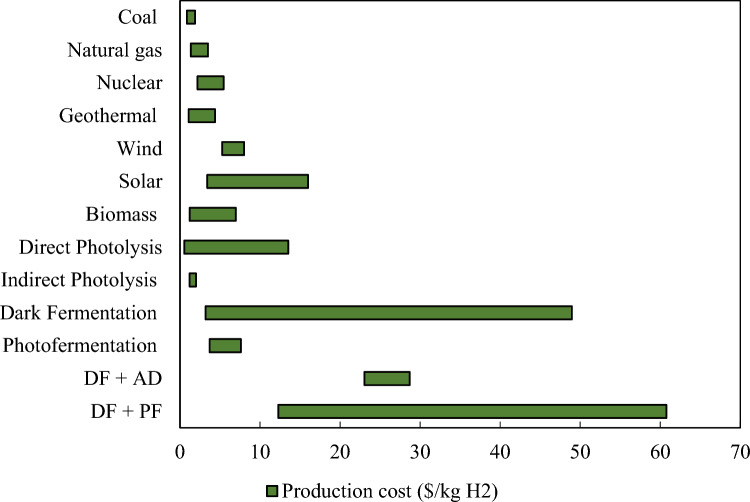
Production cost range for biological hydrogen technologies, compared to conventional coal, natural gas, and other renewable technologies. References: Biological hydrogen production pathways are provided in Table 5, differently, other production pathways production costs were sourced from Rami, (2019) (El-Emam and Özcan 2019)

Table 5 shows that various feedstock, specifically agricultural wastes, industrial waste, and food waste, could be used to produce biohydrogen through photolysis, dark fermentation, and photo-fermentation. The use of waste biomass is advantageous from a circular economy point of view as these wastes are upcycled to higher-value biofuels. Additionally, the use of waste biomass is preferred over food-competitive biomass such as corn and wheat. Although the feedstock prices are less crucial with respect overall production costs, the costs of pretreatment could be significant (Basak et al. 2020). According to Basak at el., (2020), pretreatment cost, specifically for lignocellulosic biomass contributed as high as 32% of the total production cost, a sentiment shared by the fermentative routes. Nonetheless, the feedstock price has less impact on the overall plant production cost—the critical factors are the abundance, and pretreatment strategies of the biomass to provide acceptable yields of hydrogen. On the other hand, the type of biological process significantly impacts the production costs. From Fig. 16, it is evident that hydrogen production from coal (0.86–1.89 $/kg H_2_) and natural gas (1.36–3.50 $/kg H_2_) is currently the most economically viable option. For biological hydrogen production, the cost ranges are between 0.53–13.53 $/kg bioH_2_ (direct photolysis), 3.2–48.96 $/kg bioH_2_ (dark fermentation), and 3.70–7.61 $/kg bioH_2_ (photo-fermentation). These cost ranges are for the large-scale processes provided in Table 5. The lower limits of the production cost ranges suggest that biological hydrogen production pathways could indeed be economically viable. However, there is still a large variation in the production costs regarding the higher limits, particularly, in the dark fermentation production costs.

Dark fermentation is currently the most widely investigated method with respect to TEA, mainly due to dark fermentation being a relatively mature, well-known technology compared to photo-fermentation and photolysis. It is regarded as low-cost, easy to implement industrially, and can easily be integrated into and with already existing infrastructure. Therefore, this technique is expected to be the most investigated for commercialization due to its simplicity and maturity (Lee 2015; Sillero and Gustavo 2022). Nonetheless, dark fermentation still has bottlenecks regardless of its technical and economic viability. In a study by Han (2016a, b, c), it was found that dark fermentation of food waste is economically viable with a production cost of 2.29 $/m^3^, which is lower compared to the recommended 2.7 $/m^3^. Noteworthy, the operating costs were high, with the cost of nitrogen gas used as a nutrient and purge for promoting anaerobic conditions being the highest. Similarly, in a study where wood residues from *Pinus Patula* were used to produce hydrogen through dark fermentation, it was found that the cost of raw materials (particularly enzymes used for pre-treatment) constituted a notable 64% of the total production costs. In this study, the production cost for hydrogen through dark fermentation was 35.56 $/kg bioH_2_ compared to 2–3.59 $/kg bioH_2_ for biomass gasification. Though dark fermentation was not economically attractive, with respect to environmental impact through GHG emissions, dark fermentation outperformed gasification with − 1.07 kg CO_2_/ kg *Pinus Patula* compared to + 0.30 kg CO_2_/kg *Pinus Patula* when gasified, highlighting the environmental significance of dark fermentation processes (García et al. 2017).

Photolysis and photo-fermentation are both poorly investigated in terms of technical and economic viability, with studies having adopted a theoretical approach in determining the cost of production (Dincer and Acar 2015; Sathyaprakasan and Kannan 2015; Nikolaidis and Poullikkas 2017). Both technologies demonstrate outstanding environmental impact analyses, primarily because they are not energy-intensive processes compared to dark fermentation and other renewable techniques. (Manish and Banerjee 2008) demonstrated that the GHG emissions for dark fermentation and photo-fermentation were − 87 and − 21.9 kg CO_2_/ kg bioH_2_, with dark fermentation demonstrating better environmental performance.

Indirect photolysis from microalgae production cost was estimated to be 1.2 $/ kg bioH_2_ (Anwar et al. 2019). The capital costs contributed 90% of the total costs. The production of biohydrogen from photo-fermentation and photolysis shows promise, however, the bottlenecks around this are large land requirements for the large photobioreactors, which further require significant amounts of materials, driving up the cost. Attempts to design more effectivee reactors with improved scalability have been proposed recently (Genç and Koku 2023), however, the production cost of bioH_2_ gas from molasses for this process using the PBR with 20 L capacity was 1362 $/kg bioH_2_, deeming it economically unattractive.

Both dark and photo-fermentation show promise for bioH_2_ production, with each having unique merits and shortcomings. Dark fermentation produces volatile fatty acids (VFA) and alcohols as by-products of the hydrogen process. However, the VFA are substrates to photofermentation processes, as such the by-products from the dark fermentation could be used to further enhance the hydrogen yield and process productivity through photofermentation. Apart from increasing hydrogen yield, the integrated process provides a solution for managing the organic acids waste stream. A techno-economic study by Ljunggren et al, (2011), where an integrated dark and photo-fermentation process was used to produce bioH_2_ from barley straw had an estimated production cost of 60.77 $/kg bioH_2_. The photofermentation stage in this process contributed about 90% of the total costs, through both capital investments and labor costs. The high capital cost was due to the large PBRs required both in terms of volume and area, which require vast amounts of materials (connective plastic tubing), which further required replacing annually (Ljunggren et al. 2011). The authors further highlighted that the main reason for the large photoreactors is due to the low productivity of photo fermentation (0.21 mmol H2/L/h) compared to dark fermentation (20 mmol/L/h). Undoubtedly, the productivity of photo fermentation needs to be improved to lower production costs and improve the economics of the integrated process. Additionally, a study by Urbaniec and Grabarczyk (2014) where a two-stage dark and photo-fermentation process was adopted to produce bioH2 from sugar beet molasses had high estimated production costs between 12.28–42. 13 $/kg bioH_2_. In this study, photofermentation stage costs contributed above 90% to the total production costs, this was due to high capital costs for the large PBRs needed, and thus the material costs were high (Urbaniec and Grabarczyk 2014). For production costs reduction in dark and photo-fermentation integrated process, it crucial that careful consideration for dark fermentation productivity is within reasonable operational region for photofermentation stage, else excess VFA feed would require large photoreactors to process.

Since the major drawback of the integrated dark and photo-fermentation process lies in the productivity and economics of the photofermentation stage, a logical approach would be to couple dark fermentation with anaerobic digestion (AD) to produce bioH_2_ and methane gas. Although methane gas has lower energy content compared to hydrogen, this integration still plays a role in utilising the organic waste from dark fermentation. It further increases the energy yield of the process. A study by Ljunggren and Zacchi (2010) where a two-stage dark fermentation and AD for the production of hythane (a mixture of hydrogen and methane) from potato steam starch (Ljunggren and Zacchi 2010), the estimated production cost of this process was between 7.20–48.96 $/kg biohythane. In this study, it was determined that the nutrients (yeast extract) required for both dark fermentation and AD contributes the highest toward the total production cost of the process. Interestingly, the study highlighted the low yield and productivity of integrated process has the lowest production cost of 7.20 $/kg biohythane, as this reduces the nutrient requirements. It is clear that for dark fermentation and the two-stage dark fermentation and AD process, using fewer nutrients or using less costly nutrients for the process is key to lowering the production costs. Willquist et al, (2012) conducted a techno-economic study for the production of biohythane from wheat straw (Willquist et al. 2012). It was determined that the estimated production cost is 23.04 $/kg biohythane. Similarly, the nutrient cost in this process was the major cost driver. The study highlighted that should the nutrient cost be reduced by 80%, the total production costs of the process can be reduced by 44%. Noteworthy, this study produces biohythane and not bioH_2_, as such the production costs should increase when hydrogen is recovered from the biohythane mixture.

## Future perspectives

Biohydrogen relies heavily upon large-scale production and economic viability to sustainably meet demands. However, certain barriers pertaining to the (1) biohydrogen production strategies, (2) biohydrogen production systems, (3) modelling of the biohydrogen production systems, and (4) the techno-economic and life cycles assessment, for decision making currently hamper the field.

### Biohydrogen production strategies

Biohydrogen production strategies discussed in Sect. “Biological hydrogen production strategies” are inherently associated with specific and/or competing advantages, and challenges. Table 6 summarizes these advantages and challenges.

**Table 6:** Main advantages and challenges associated with the different biological hydrogen production strategies

Strategy	Advantages	Challenges
Direct Biophotolysis	No nutrient requirements	H_2_ yield limited by oxygen-sensitivity of hydrogenase enzyme
	Only requires water and light	Low light conversion efficiency
		Requires a light source
Photofermentation	Not oxygen-evolving	Requires anaerobic conditions
	Purple bacteria can utilize a wide light emission spectrum	Requires large surface-to-volume ratio in reactors
	Organic carbon-rich waste streams can be utilized as substrate	Requires a light source
	High carbon to H_2_ conversion yield	Waste stream substrates require pre-treatment
		Contamination hampers H_2_ production
		Low light conversion efficiency
		Low hydrogen production rates
Dark Fermentation	Light-independent	Generates biogas as by-product
	Able to utilize a wide range of carbon substrates	Evolved gas requires downstream purification due to the presence of CO_2_ etc
	High H_2_ production rates	Generates large quantities of organic by-products
	Organic carbon-rich waste streams can be utilized as substrate	Oxygen sensitive
		Low hydrogen yield
		Thermodynamic limitations on H_2_ production

When analyzing the future perspectives of these production strategies, different strategies are at different levels of development towards scalability. However, dark fermentation appears to be the preferred route. Unfortunately, there are scarce literature studies examining the feasibility of outdoor photofermentation and/or scaled-up operations. Thus, a significant need exists for research into the viability of upscaling such biohydrogen production processes. Ultimately, the economic feasibility and the corresponding need for pre-treatment of suitable waste streams—a factor which seems to be critical in the view of potential industrialization of both photo- as well as dark fermentation processes.

### Biohydrogen reactor mode of operation and the effect of configuration

Regarding the three traditional batch, fed batch and continuous modes of biohydrogen production reviewed in the Modes of operation used for biohydrogen production section, the batch modes were dominantly used for both dark and/or photo- fermentations. Although out scalable in modular units, if the economies of scale is to benefit the process, then the latter continuous or fed-batch modes of operation are preferred as production yield could be increased as compared to the batch mode. Therefore, industrial prospects require transferring from laboratory scale under batch fermentation, to long-term continuous or fed-batch large scale production.

With regards to bioreactor configuration, different configurations have been tested in the literature. However, no specific configuration clearly outperforms all others yet. Finally, the combination of different configurations, has not adequately been investigated, with limited studies focusing on multistep processes.

### Modelling of biohydrogen production systems

The advent of high-performance computing resources and advanced mathematical theories (e.g., for optimization, and statistical analysis), unlocks the potential for simulating large-scale biohydrogen production. Although the state-of-the-art revealed promising modelling techniques in the Modelling of biohydrogen production systems section, advances in (1) computational strain design (i.e., metabolic engineering), (2) integration of CFD solvers with MFA models, and (3) exploitation of other machine learning algorithms (e.g., Gaussian process) and the biohydrogen uncertainties quantification, could improve the current biological barriers. Moreover, algorithms for optimal design of computational strains could embed bi-level and mixed integer programming frameworks to mathematically deactivate redundant fluxes and maximize the biohydrogen flux. As bioreactor mixing is expected to consequentially effect the metabolic flux distributions via hydrodynamic induced shear stress, CFD solvers integrating MFA models do hold strong potential for optimizing both the mixing energy and bioreactor operating conditions. Typical of all bioprocesses, biohydrogen production is no exemption to the associated large uncertainties (e.g., batch-to-batch variations). Therefore, machine learning models like Gaussian process have potential for robust biohydrogen modelling and decision making under uncertainty.

### Techno-economic and life cycle assessment strategies

TCAs coupled with LCAs are crucial for a comprehensive understanding of the technical, economic, and environmental aspects of biohydrogen production processes. Although bioH_2_ process production costs are still high and economically unattractive, these costs are expected to decline as technology advances. It is clear that dark fermentation is currently the route with high potential for commercialization, however, efforts and research need to be concentrated on lowering raw materials costs (specifically added nutrients). The shortcomings of both photolysis and photo fermentation processes are undoubtedly the high capital and operating costs associated with the PBRs. Research towards increasing the productivity of PBRs is crucial in reducing production costs for these processes. Integrated approaches, such as combining dark fermentation with photo fermentation or anaerobic digestion (AD), can enhance the overall process efficiency and energy yield. The integration of dark fermentation and AD for biohydrogen and methane production (biohythane) shows potential, although the productivity and economics of the photofermentation stage need improvement. By concentrating efforts on reducing the cost drivers for bioH_2_, it can realize its full commercialization potential as a cleaner and sustainable alternative to fossil fuels.

## Conclusions

This review provides a summary of research in the field of biohydrogen production, demonstrating future prospects of industrial importance. It is evident that the field of biohydrogen is growing with a wide range of applications, particularly in waste conversion, and methodologies for hydrogen production are actively under investigation by researchers.. Hence, realistic operational processes for scale up capabilities required further demonstration as discussed below.

*Exploration on the production strategy, their operational modes and the producing systems* In this review a summary of the biohydrogen production routes are given, as well as the producing mode of operations and bioreactor configurations summarized. Although these aspects have demonstrated biohydrogen production, the production strategy and modes of operations are at different levels of development with batch mode and dark fermentation more dominant in the literature. However, for scalability purposes, fed batch and continuous production have advantages, while pretreatment strategies depending on the utilized substrate and could be investigated to further increase yield and productivities. Also, more emphasis could be assigned on investigating outdoor production of biohydrogen, with the significant increase in the use of photo-fermentation. More investigation on the combination of producing strategies (biophotolysis, dark-, and photo-fermentation) are required, as they will allow for industrial symbiosis of the technology and open room for more dynamic models that could facilitate scalability and enhance yield increments.

*Scalability potential* although this review touches on the methods and options for modelling these complex processes, critical understanding of the different system dynamics and hydrodynamics are required. Should this be available, scaled-up production will be facilitated through application of CFD modelling and the machine learning algorithms which are rapidly developing. Since more research is needed in the photo-fermentation sphere, proper incorporation of the lighting aspects for scale-up could play a crucial role in developing even larger production units for outdoor system.

*Economies of scale* with the techno-economic understanding of biohydrogen production, economies of scale in conjunction will life-cycle assessment will be vital to understand the technical, economic, and environmental aspects of proposed processes.

While there is significant work presented in the literature on biological hydrogen production, it is evident that there is still much work needed to scale-up processes and develop technologies so that biological hydrogen can be cost-competitive with current hydrogen production routes.

## Data Availability

No data were generated in this article. Data summarized in tables are drawn from the relevant references and can be accessed there.
